# Recovery schools for improving behavioral and academic outcomes among students in recovery from substance use disorders: a systematic review

**DOI:** 10.4073/csr.2018.9

**Published:** 2018-10-04

**Authors:** Emily A. Hennessy, Emily E. Tanner‐Smith, Andrew J. Finch, Nila Sathe, Shannon Kugley

## Abstract

This Campbell systematic review examines the effects of recovery schools on student behavioral and academic outcomes, compared to the effects of non‐recovery schools. The review summarizes evidence from one quasi‐experimental study (with a total of 194 participants) that had potential serious risk of bias due to confounding.

Sizable portions of youth are in recovery from substance use disorders, and many youth will return to use after receiving substance use treatment. Youth spend most of their waking hours at school, and thus schools are important social environments for youth in recovery from substance use disorders. Recovery schools have been identified as educational programs that may help support youth in recovery from substance use disorders.

This review focused on two types of recovery schools: RHSs, which are schools that award secondary school diplomas and offer a range of therapeutic services in addition to standard educational curricula; and CRCs, which offer therapeutic and sober support services on college campuses.

This review looked at whether recovery schools (RHSs or CRCs) affect academic success and substance use outcomes among students, compared to similar students who are not enrolled in recovery schools.

**Plain language summary:**

**There is insufficient evidence to know whether recovery high schools and collegiate recovery communities are effective**

Evidence that recovery high schools (RHSs) may improve academic and substance use outcomes is based on the findings from a single study with a serious risk of bias.

**The review in brief**

Very limited evidence addresses the effectiveness of recovery high schools (RHSs). There is no rigorous evidence on the effectiveness of collegiate recovery communities (CRCs).

It is unclear whether CRCs are effective in promoting academic success and reducing substance use among college students.

**What is this review about?**

Based on the results of one study, RHSs may reduce high school students' school absenteeism, marijuana use, and other drug use, and increase abstinence from drugs; but RHSs may be no better or worse than other high schools in improving grades, reducing truancy, or reducing alcohol use.

**What is the aim of this review?**

This Campbell systematic review examines the effects of recovery schools on student behavioral and academic outcomes, compared to the effects of non‐recovery schools. The review summarizes evidence from one quasi‐experimental study (with a total of 194 participants) that had potential serious risk of bias due to confounding.

**What are the main findings of this review?**

Sizable portions of youth are in recovery from substance use disorders, and many youth will return to use after receiving substance use treatment. Youth spend most of their waking hours at school, and thus schools are important social environments for youth in recovery from substance use disorders. Recovery schools have been identified as educational programs that may help support youth in recovery from substance use disorders.

This review focused on two types of recovery schools: RHSs, which are schools that award secondary school diplomas and offer a range of therapeutic services in addition to standard educational curricula; and CRCs, which offer therapeutic and sober support services on college campuses.

This review looked at whether recovery schools (RHSs or CRCs) affect academic success and substance use outcomes among students, compared to similar students who are not enrolled in recovery schools.

**What studies are included?**

The included study of recovery high schools used a controlled quasi‐experimental pretest‐posttest design and reported on the following outcomes: grade point average, truancy, school absenteeism, alcohol use, marijuana use, other drug use, and abstinence from alcohol/drugs. The included study focused on a sample of U.S. high school students. There were no eligible studies of CRCs.

**What do the findings of this review mean?**

Findings from this review indicate insufficient evidence on the effects of recovery schools on student well‐being. Although there is some indication RHSs may improve academic and substance use outcomes, this is based on the findings from a single study. There is no available evidence on the effects of CRCs.

No strong conclusions can be drawn at this time, given the lack of available evidence on RHSs and CRCs, and the serious risk of bias in the one RHS study included in the review. The evidence from this review suggests there is a clear need for additional rigorous evaluations of recovery school effects prior to widespread implementation.

**How up‐to‐date is this review?**

The review authors searched for studies until September 2018. This Campbell systematic review was published in 2018.

**Executive Summary/Abstract:**

**BACKGROUND**

Substance use disorders (SUDs) among youth are a major public health problem. In the United States, for example, the incidence of SUDs increases steadily after age 12 and peaks among youth ages 18–23 (White, Evans, Ali, Achara‐Abrahams, & King, 2009). Although not every youth who experiments with alcohol or illicit drugs is diagnosed with an SUD, approximately 7–9% of 12–24 year olds in the United States were admitted for public SUD treatment in 2013 (Substance Abuse and Mental Health Services Administration [SAMHSA], 2016). Recovery from an SUD involves reduction or complete abstinence of use, defined broadly as “voluntarily sustained control over substance use, which maximises health and wellbeing and participation in the rights, roles and responsibilities of society” (UK Drug Policy Commission, 2008). However, SUDs are often experienced as chronic conditions; among youth who successfully complete substance use treatment, approximately 45–70% return to substance use within months of treatment discharge (Anderson, Ramo, Schulte, Cummins, & Brown, 2007; Brown, D'Amico, McCarthy, & Tapert, 2001; Ramo, Prince, Roesch, & Brown, 2012; White et al., 2004). Thus, multiple treatment episodes and ongoing recovery supports after treatment are often necessary to assist with the recovery process (Brown et al., 2001; Ramo et al., 2012; White et al., 2004).

Success and engagement at school and in postsecondary education are critical to healthy youth development. For youth in recovery from SUDs, school attendance, engagement, and achievement build human capital by motivating personal growth, creating new opportunities and social networks, and increasing life satisfaction and meaning (Keane, 2011; Terrion, 2012; 2014). Upon discharge from formal substance use treatment settings, schools become one of the most important social environments in the lives of youth with SUDs. Healthy school peer environments can enable youth to replace substance use behaviors and norms with healthy activities and prosocial, sober peers. Conversely, many school environments may be risky for youth in recovery from SUDs due to perceived substance use among peers, availability of drugs or alcohol, and substance‐approving norms on campus (Centers for Disease Control [CDC], 2011; Spear & Skala, 1995; Wambeam, Canen, Linkenbach, & Otto, 2014).

Given the many social and environmental challenges faced by youth in recovery from substance use, recovery‐specific institutional supports are increasingly being linked to educational settings. The two primary types of education‐based continuing care supports for youth in recovery, defined under the umbrella term of “recovery schools” for this review, are recovery high schools (RHSs) and collegiate recovery communities (CRCs). RHSs are secondary schools that provide standard high school education and award secondary school diplomas, but also include therapeutic programming aimed at promoting recovery (e.g., group check‐ins, community service, counseling sessions). CRCs also provide recovery oriented support services (e.g., self‐help groups, counseling sessions, sober dorms) for students, but are embedded within larger college or university settings. The primary aims of RHSs and CRCs are to promote abstinence and prevent relapse among students, and thus ultimately improve students' academic success.

**OBJECTIVES**

This review summarized and synthesized the available research evidence on the effects of recovery schools for improving academic success and behavioural outcomes among high school and college students who are in recovery from substance use. The specific research questions that guided the review are as follows:

1. What effect does recovery school attendance (versus attending a non‐recovery or traditional school setting) have on academic outcomes for students in recovery from substance use? Specifically (by program type):
  a. For recovery high schools: what are the effects on measures of academic achievement, high school completion, and college enrolment?
  b. For collegiate recovery communities: what are the effects on measures of academic achievement and college completion?
2. What effect does recovery school attendance have on substance use outcomes for students in recovery from substance use? Specifically, what are the effects on alcohol, marijuana, cocaine, or other substance use?
3. Do the effects of recovery schools on students' outcomes vary according to the race/ethnicity, gender, or socioeconomic status of the students?
4. Do the effects of recovery schools on students' outcomes vary according to existing mental health comorbidity status or juvenile justice involvement of the students?

**SEARCH METHODS**

We aimed to identify all published and unpublished literature on recovery schools by using a comprehensive and systematic literature search. We searched multiple electronic databases, research registers, grey literature sources, and reference lists from prior reviews; and contacted experts in the field.

**SELECTION CRITERIA**

Studies were included in the review if they met the following criteria:

**Types of studies:** Randomized controlled trial (RCT), quasi‐randomized controlled trial (QRCT), or controlled quasi‐experimental design (QED).

**Types of participants:** Students in recovery from substance use who were enrolled part‐time or full‐time in secondary (high school) or postsecondary (college or university) educational institutions.

**Types of interventions:** Recovery schools broadly defined as educational institutions, or programs at educational institutions, developed specifically for students in recovery and that address recovery needs in addition to academic development.

**Types of comparisons**: Traditional educational programs or services that did not explicitly have a substance use recovery focus.

**Types of outcome measures:** The review focused on primary outcomes in the following two domains: academic performance (e.g., achievement test scores, grade‐point average, high school completion, school attendance, college enrolment, college completion) and substance use (alcohol, marijuana, cocaine, heroin, stimulant, mixed drug use, or other illicit drug use). Studies that met all other eligibility criteria were considered eligible for the narrative review portion of this review even if they did not report outcomes in one of the primary outcome domains.

**Other criteria:** Studies must have been reported between 1978 and 2016. The search was not restricted by geography, language, publication status, or any other study characteristic.

**DATA COLLECTION AND ANALYSIS**

Two reviewers independently screened all titles and abstracts of records identified in the systematic search. Records that were clearly ineligible or irrelevant were excluded at the title/abstract phase; all other records were retrieved in full‐text and screened for eligibility by two independent reviewers. Any discrepancies in eligibility assessments were discussed and resolved via consensus. Studies that met the inclusion criteria were coded by two independent reviewers using a structured data extraction form; any disagreements in coding were resolved via discussion and consensus. If members of the review team had conducted any of the primary studies eligible for the review, external and independent data collectors extracted data from those studies. Risk of bias was assessed using the ROBINS‐I tool for non‐randomized study designs (Sterne, Higgins, & Reeves, 2016).

Inverse variance weighted random effects meta‐analyses were planned to synthesize effect sizes across studies, as well as heterogeneity analysis, subgroup analysis, sensitivity analysis, and publication bias analysis. However, these synthesis methods were not used given that only one study met the inclusion criteria for the review. Instead, effect sizes (and their corresponding 95% confidence intervals) were reported for all eligible outcomes reported in the study.

**RESULTS**

Only one study met criteria for inclusion in the review. This study used a QED to examine the effects of RHSs on high school students' academic and substance use outcomes. No eligible studies examining CRCs were identified in the search.

The results from the one eligible RHS study indicated that after adjusting for pretest values, students in the RHS condition reported levels of grade point averages (= 0.26, 95% CI [‐0.04, 0.56]), truancy (= 0.01, 95% CI [‐0.29, 0.31]), and alcohol use (= 0.23, 95% CI [‐0.07, 0.53]) similar to participants in the comparison condition. However, students in the RHS condition reported improvements in absenteeism (= 0.56, 95% CI [0.25, 0.87]), abstinence from alcohol/drugs (*OR* = 4.36, 95% CI [1.19, 15.98]), marijuana use (= 0.51, 95% CI [0.20, 0.82]), and other drug use (= 0.45, 95% CI [0.14, 0.76]).

Overall, there was a serious risk of bias in the one included study. The study had a serious risk of bias due to confounding, low risk of bias due to selection of participants into the study, moderate risk of bias due to classification of interventions, inconclusive risk of bias due to deviations from intended interventions, inconclusive risk of bias due to missing data, moderate risk of bias in measurement of outcomes, and low risk of bias in selection of reported results.

**AUTHORS’ CONCLUSIONS**

There is insufficient evidence regarding the effectiveness of RHSs and CRCs for improving academic and substance use outcomes among students in recovery from SUDs. Only one identified study examined the effectiveness of RHSs. Although the study reported some beneficial effects, the results must be interpreted with caution given the study's potential risk of bias due to confounding and limited external validity. No identified studies examined the effectiveness of CRCs across the outcomes of interest in this review, so it is unclear what effects these programs may have on students' academic and behavioral outcomes.

The paucity of evidence on the effectiveness of recovery schools, as documented in this review, thus suggest the need for caution in the widespread adoption of recovery schools for students in recovery from SUDs. Given the lack of empirical support for these recovery schools, additional rigorous evaluation studies are needed to replicate the findings from the one study included in the review. Furthermore, additional research examining the costs of recovery schools may be needed, to help school administrators determine the potential cost‐benefits associated with recovery schools.

## 1 Background

### 1.1 THE PROBLEM, CONDITION OR ISSUE

#### 1.1.1 Youth substance use disorders

Substance use disorders (SUDs) among youth are a major public health problem. In the United States, for example, the incidence of SUDs increases steadily after age 12 and peaks among youth ages 18–23 (White et al., 2009). Although not every youth who experiments with alcohol or illicit drugs is diagnosed with an SUD, approximately 7–9% of 12–24 year olds in the United States were admitted for public SUD treatment in 2013 (SAMHSA, 2016). The true prevalence of SUDs among youth in the United States is likely higher than 7–9%, however, given that many youth receive treatment in private or in non‐specialty settings (Laudet, Harris, Kimball, Winters, & Moberg, 2014), and other youth may never receive treatment for their substance use problems (SAMHSA, 2015). Data available on substance use patterns and treatment availability in other nations also suggests that there are significant numbers of youth worldwide in need of some form of substance use treatment and/or aftercare, although this research is primarily limited to developed nations. For example, in Australia, adolescents aged 10–19 years old comprised approximately 12% of all treatment admissions from 2012–2013 (Australian Institute of Health and Welfare, 2014). Similarly, the European Council estimated that across surveyed countries, youth constituted anywhere from 16% (Italy) to 65% (Czech Republic) of the overall substance use treatment population (Council of Europe, Pompidou Group, 2006). And, in 2011, approximately 28,000 adolescents were newly admitted to outpatient treatment across Europe (European Monitoring Centre for Drugs and Drug Addiction, 2013).

Substance use problems can have numerous detrimental consequences on the academic, social, and general well‐being of youth. This might include negative effects on school or work performance, legal problems, and substance use tolerance and progression (American Psychiatric Association [APA], 2013). Prior research has documented the multiple negative effects from prolonged and heavy substance use, including diminished memory and cognitive abilities, reduced grades, a decreased likelihood of finishing high school or attending post‐secondary education, problems attaining or keeping employment, higher rates of more acute and chronic health conditions than those without a history of use, poverty, and family and social problems (Brown & Tapert, 2004; Homel, Thompson, & Leadbeater, 2014; Larm, Hodgins, Larsson, Samuelson, & Tengström, 2008; Leslie et al., 2016; Lisdahl, Wright, Medina‐Kirchner, Maple, & Shollenbarger, 2014; Menasco & Blair, 2014; Newcomb & Bentler, 1988; Patrick, Schulenberg, & O'Malley, 2016; Silins et al., 2014; Squeglia, Jacobus, & Tapert, 2009; Thoma et al., 2011). Given the numerous negative effects associated with heavy substance use among youth, it is important to understand what programs and interventions might be effective in assisting youth with SUDs.

#### 1.1.2 Youth development, problematic substance use, and substance use disorders

The period of adolescence “is characterized by more biological, psychological, and social role changes than any other stage of life except infancy” (Holmbeck, Devine, Wasserman, Schellinger, & Tuminello, 2012, p. 431). These changes begin in adolescence and continue into emerging adulthood and involve pubertal and cognitive development, and identity, relationship, and achievement transitions (Schulenberg & Maggs, 2001). Generally, the period of adolescence lasts from ages 10–19, although the World Health Organization's term “young people” includes individuals between 10–24 years of age (World Health Organization, 2016).

Adolescent neurodevelopment has been described as a biologically critical period of heightened vulnerability to experimentation and habitual use of substances (Chambers, Taylor, & Potenza, 2003). For example, due to neurodevelopmental processes, adolescents are more prone to impulsive behaviors, risk taking, and drug and reward seeking compared to adults—all of which are behaviors that are linked to substance use (Chambers et al., 2003; Simon & Moghaddam, 2015). Indeed, animal studies of neural processing have demonstrated that adolescent and adult brains respond differently to rewards, with activity in adolescents' brain regions of learning and habit formation potentially predisposing them to form problematic substance use habits (Sturman & Moghaddam, 2012). This area of research has also demonstrated that adolescents consume larger quantities of alcohol than adults, a behavior that continues into adulthood, suggesting that drinking habits begun early in life, i.e., during adolescence, may worsen relative to habits begun during adulthood and extend for longer periods of time (Serlin & Torregrossa, 2015).

In addition to neurodevelopmental findings, research has demonstrated that adolescents develop expectancies about potential benefits or risks of substance use (Schulenberg & Maggs, 2001); these expectancies can vary depending on adolescents' social networks and settings, and affect when and how they decide to use substances. The social context is especially important as adolescents undergo relationship changes with parents by seeking to differentiate themselves as autonomous individuals and youth create relationships with peers that become more influential. Youth also tend to overestimate the prevalence of drinking in their social environment, with college students viewing drinking as highly normative; youth may also experience increased sociability and report better bonding with peers as a result of drinking (Schulenberg & Maggs, 2001). Social influence has also been consistently identified as a predictor of return to substance use among recovering adolescents (Ramo et al., 2012).

In addition, specific changes related to this developmental period have been identified as risk factors for more problematic substance use (e.g., early maturation, affiliation with deviant peers, and conflicts with parents; Weisz & Hawley, 2002). Adolescent development processes as well as the presence of these additional factors are both risk factors for problematic use, setting this population apart from adults and thus, treatments and aftercare supports should take developmental issues into account (Deas, Riggs, Langenbucher, Goldman, & Brown, 2000; Weisz & Hawley, 2002). In support of this contention, addiction literature has documented different substance use precipitants for adolescents than for adults, with adolescents more likely to return to use under social pressure and also exhibiting greater complexity in these patterns (Ramo & Brown, 2008). Adolescents often use multiple substances and may use a broader array of substances than adults (Deas et al., 2000).

Although not all experimentation with substances leads to problematic use, problematic use can transition to a substance use disorder (SUD), i.e., “a maladaptive pattern of substance use leading to clinically significant impairment or distress” (APA, 1994). Substance abuse and dependence were originally diagnosed separately with substance abuse considered an early phase and dependence the more severe manifestation; however, the Diagnostic and Statistical Manual of Mental Disorders‐V (DSM‐V) modified the definition of an SUD to combine abuse and dependence into one category with different levels (APA, 2013). An SUD is manifested in multiple ways, including substance use that repeatedly impacts school or work performance or contributes to legal problems. Individuals with an SUD demonstrate an increasing need for more of the substance to achieve the same effect (i.e., tolerance) and taking the substance for longer or in larger doses and experience unsuccessful attempts to quit using the substance. Thus, recovery from an SUD is a process involving many factors, the hallmark of which is reduction or complete abstinence of use. Recovery also refers to overall healthy functioning and has been defined as “voluntarily sustained control over substance use, which maximises health and wellbeing and participation in the rights, roles and responsibilities of society” (UK Drug Policy Commission, 2012).

### 1.2 THE INTERVENTION

#### 1.2.1 Approaches for addressing youth in recovery from substance use disorders

Given the biological, psychological, and social developmental changes in adolescence, it is important to attend to their distinct developmental issues when focusing on adolescents in recovery (Weisz & Hawley, 2002). Thus, for youth diagnosed with an SUD there are a variety of adolescent‐specific treatment options available, which fall within a spectrum of varying intensity from early intervention, such as screening, brief intervention, and referral to treatment (SBIRT: Bien, Miller, & Tonigan, 1993; Madras et al., 2009) to intensive inpatient treatment (American Society of Addiction Medicine [ASAM], 2013). However, SUDs are often experienced as chronic conditions; thus, multiple treatment episodes and ongoing recovery supports after treatment are often necessary to assist with the recovery process (Brown et al., 2001; Ramo et al., 2012; White et al., 2004). Indeed, research has demonstrated that youth seeking SUD treatment do not always engage in or successfully complete treatment (Kaminer, Burleson, Burke, & Litt, 2014; Pugatch, Knight, McGuiness, Sherritt, & Levy, 2014; Winters, Stinchfield, Latimer, & Lee, 2007). Lack of engagement in treatment is due to a variety of factors including denial about the extent of the problem, motivation, emotional reasons, life stressors, financial or insurance barriers, peer influence and social norms, and access and availability of substances combined with triggers to use (Gonzales, Anglin, Beattie, Ong, & Glik, 2012; Wisdom, Cavaleri, Gogel, & Nacht, 2011), and has been linked to community factors such as median family income (Jones, Heflinger, & Saunders, 2007). Among youth who successfully complete substance use treatment programs, 45–70% return to substance use within months of treatment discharge (Anderson et al., 2007; Brown, et al., 2001; Ramo et al., 2012; White et al., 2004).

Perhaps the greatest risk of relapse for youth in recovery is during the late adolescent and emerging adulthood years (Anderson, Ramo, Cummins, & Brown, 2010); thus, youth with an SUD require developmentally appropriate, sustained, and multi‐pronged intervention and follow‐up support (Gonzales et al., 2012). To this end, research has demonstrated the importance of structured continuing care supports after treatment for youth in recovery from substance use. These continuing care supports can include, for instance, a dedicated case manager, home visits, meetings with caregivers, or other environmental supports for the youth and their family (Godley et al., 2014; Stanger, Ryan, Scherer, Norton, & Budney, 2015; Tanner‐Smith, Wilson, & Lipsey, 2013). Indeed, youth who engage in recovery supports posttreatment have the greatest likelihood of abstinence from substance use (Brown et al., 2001; Hennessy & Fisher, 2015). Engagement in substance‐free peer environments is one recovery support system that shows particular promise, and has been linked to reduced substance use and increased psychosocial functioning among youth (Anderson, Ramo, Schulte, Cummins, & Brown, 2008; Nelson, Henderson, & Lackey, 2015; Terrion, 2012).

#### 1.2.2 The importance of schools in the recovery process

Success and engagement at school and in postsecondary education are critical to healthy youth development. For youth in recovery from SUDs, school attendance, engagement, and achievement build human capital by motivating personal growth, creating new opportunities and social networks, and increasing life satisfaction and meaning (Keane, 2011; Terrion, 2012; 2014). Upon discharge from formal substance use treatment settings, schools become one of the most important social environments in the lives of youth with SUDs. Healthy school peer environments can enable youth to replace substance use behaviors and norms with healthy activities and prosocial, sober peers.

Unfortunately, however, some of the most significant risk factors for substance use are embedded within school environments, including perceived peer use, association with substance‐using peers, alcohol or drug availability, and stressors such as academic challenges (Derzon, 2007; Mason, Mennis, Linker, Bares, & Zaharakis, 2014; Svensson, 2000; Wambeam et al., 2014). Indeed, in a nationwide survey of high school students in the United States, approximately 26% of respondents were offered, sold, or given an illicit drug on school property (CDC, 2012). This trend is especially problematic for youth in recovery from SUDs: for example, in a study of recovering youth, almost all adolescents who returned to their old school after treatment reported being offered drugs on their first day back in high school (Spear & Skala, 1995). College students suffer similar environmental risks, particularly given the high rates of, and social norms that approve of alcohol consumption on campus. For example, a study of seven universities in Great Britain demonstrated that approximately 70% of enrolled students reported heavy drinking at least once during the previous two weeks. Across five New Zealand universities, 37% of student respondents reported at least one binge drinking episode during the previous week and approximately 68% scored in the hazardous drinking range on the Alcohol Use Disorders Identification Test consumption scale (Kypri et al., 2009). In the United States, 35–39% of college students reported binge drinking (i.e., five/four or more drinks in one sitting for males/females, respectively) at least once in the past month (Monitoring the Future, 2013; SAMHSA, 2014) and research has documented a rapid escalation in alcohol consumption during the first two years of college in the United States (Schulenberg & Maggs, 2001). Indeed, approximately one‐half of SUD treatment admissions for college students in the United States were for alcohol use (Center for Behavioral Health Statistics and Quality, 2012). In addition, a recent review highlighted that approximately 20% of college students in the United States use drugs on a monthly basis and that 5% reported daily use (Dennhardt & Murphy, 2013). One study of recovering college students found that the majority of students were addicted to multiple substances while only a few students were solely addicted to alcohol (Cleveland, Harris, Baker, Herbert, & Dean, 2007). Given the prevalence of binge drinking and substance use among this population as well as the social acceptability of alcohol consumption during the college years, it is no surprise that the college environment has been described as “abstinence hostile” for youth in recovery (Cleveland et al., 2007).

#### 1.2.3 Recovery schools as interventions to improve students' well‐being

Given the many social and environmental challenges faced by youth in recovery from substance use, recovery‐specific institutional supports are increasingly being linked to educational settings. The two primary types of education‐based continuing care supports for youth in recovery are recovery high schools (RHS) and collegiate recovery communities or programs (CRC): both settings will be referred to under the broad umbrella term of “recovery schools” for this review. Federal offices in the United States recognize these two educational programs as viable supports for youth after they complete formal substance use treatment programs (National Institute on Drug Abuse [NIDA], 2014; Office on National Drug Control and Policy [ONDCP], 2014).

##### 1.2.3.1 Recovery high schools

The RHS model addresses academic advancement and recovery maintenance among adolescents that have completed treatment for an SUD and are still seeking to complete their high school education (Finch & Frieden, 2014). First instituted in 1979, RHSs are now located in multiple cities across the United States and are typically small, with school size on average around 30–40 students (Ruben, 2000; White & Finch, 2006). According to the Association of Recovery Schools (ARS), a national United States‐based non‐profit organization that supports the creation, improvement, and maintenance of RHSs, there are currently 36 recovery high schools in operation in the United States (ARS, 2016), although there have been as many as 84 operating over the past 30 years (Finch, Karakos, & Hennessy, 2016). Depending on the location and policies of the educational system in which it is embedded, an RHS may be free of charge for students or provide scholarships for tuition (Finch et al., 2016). Although the ARS has recently developed accreditation standards and begun to review RHSs under these standards, RHSs are implemented using a variety of models and share a set of common characteristics (Finch, Moberg, & Krupp, 2014). The RHS's primary purpose is to educate youth in recovery from substance use or co‐occurring disorders (ARS, 2013). Thus, RHSs have two primary foci: (1) an educational focus, which includes meeting state requirements for awarding a secondary school diploma; and (2) a recovery focus that ensures that all students enrolled at the school are in recovery for substance use or co‐occurring disorders and that they work toward maintaining recovery while enrolled. Finally, RHSs should be available to any student who is in recovery and who would meet state or district eligibility for attendance.

Academics are a primary focus in RHSs, however, the schools also incorporate recovery‐specific elements into the day, such as a daily group check‐in, community service, and individual counseling sessions. Schools employ administrative and teaching staff, but vary in their employment of counseling staff. For example, some schools have dedicated counselors located daily at the school, while others employ mental health staff to visit the school on a regular basis for individual meetings with students (Finch et al., 2014). Most schools have admission criteria that require some SUD treatment and/or minimum length of sobriety and a desire to remain abstinent; however, if a school is part of the public school system, they are unable to mandate previous treatment for enrolling students and instead rely on interviews with youth to determine their interest in maintaining sobriety and contributing to the sober culture (Finch & Wegman, 2012). In addition, RHSs encourage parent involvement in their students' learning experience through the creation of parent support groups (Finch et al., 2014) and some schools mandate this practice via regular parent‐teacher meetings. These criteria, along with structured and supervised learning, foster a recovery‐supportive culture where students and staff attend to academic development and recovery maintenance. Thus, the characteristics that distinguish RHSs from traditional high schools include attention to maintenance of a positive, sober peer culture and explicit therapeutic support (Tanner‐Smith, Finch, Moberg, & Karakos, 2014). Because peer substance use is highly predictive of one's own substance use (Lewis & Mobley, 2010), the RHS sober peer environment is especially important to the RHS model.

Descriptive research has demonstrated that students in RHSs do feel supported by peers in the RHS setting (Karakos, 2014), and that RHSs may be effective in promoting the health and well‐being of students. For example, one evaluation of three RHSs in Massachusetts found that the majority of students remained enrolled in the school for the year during the study and maintained A or B grades while also remaining abstinent or only enduring 1–2 relapse episodes during this year; Interviews with youth also demonstrated psychosocial benefits to remaining in the RHS (Kochanek, 2008). Another descriptive pre‐post study of 17 RHSs found that students enrolled in these schools had reduced substance use and improved mental health functioning at follow‐up (Moberg & Finch, 2008).

##### 1.2.3.2 Collegiate recovery communities

As a result of the substance use environment on college campuses, CRCs have been instituted with college/university support or via grassroots student organizations on at least 75 college campuses to provide college students with recovery supports and a social environment that encourages abstinence (Bugbee, Caldeira, Soong, Vincent, & Arria, 2016; Harris, Kimball, Casiraghi, & Maison, 2014). Recent data suggest that as many as 600 students are members of CRCs in the United States every academic year (Laudet et al., 2014). Given that students are typically members of CRCs for a relatively short amount of time (i.e., their duration in college), there is a likely possibility that many college enrolled youth in recovery spend time in these communities; thus, the number of unique student members is likely much higher than 600.

Similar to RHSs, CRCs are typically small communities and range in size from 5–80 students (Cleveland et al., 2007). The CRC model focuses heavily on providing environmental sober supports and settings and incorporates recovery‐related supports such as seminars, 12‐Step meetings and counseling, recreational activities and social events, life‐skills training, educational supports and peer and family support, as well as linkages to community supports and services (Bugbee et al., 2016; Harris, Baker, Kimball, & Shumway, 2008; Laudet et al., 2014). Some CRCs also require that members sign a behavioral and sobriety contract that may be implemented and enforced by a peer governance system (Bugbee et al., 2016). Similar to RHSs, some CRCs also have admission criteria for their community programs. For example, one CRC at a large public university requires that members have been in recovery for one year, continue to pursue their education, and are willing to attend at least one on‐campus 12‐Step meeting each week (Cleveland et al., 2007). Other CRCs may require that student members have previously received treatment for addiction.

CRCs differ from RHSs, however, in that although they offer educational supports, they do not usually offer separate academic classes for students in recovery. They primarily provide recovery support services including offering group meetings and individual counseling services, providing dedicated space for students in recovery to meet, organizing sober events and community service activities, and educating the broader community in an effort to reduce stigma around addiction (Association of Recovery in Higher Education [ARHE], 2015; Cleveland et al., 2007; Harris et al., 2014; Perron et al., 2011). They also may offer scholarships toward university fees and provide substance‐free, supervised dormitories (Cleveland et al., 2007; Harris et al., 2014), although presence of a sober dormitory does not in itself constitute a CRC (Bugbee et al., 2016).

The primary aim of the CRC is to improve abstinence rates and prevent relapse so that students can remain enrolled and succeed at secondary education (Harris et al., 2014): indeed, preliminary descriptive research demonstrates evidence of this success (Cleveland et al., 2007). For example, the relapse rate among CRC members across one academic semester was only 4.4%. In addition, the majority of CRC members in this study had solid academic records while enrolled at the university and many maintained working part/full time jobs. And, in a nationwide survey of students enrolled in 29 different CRCs, approximately only 5% of students who considered themselves in recovery from addiction had used a substance in the past month (Laudet, Harris, Kimball, Winters, & Moberg, 2016).

### 1.3 HOW THE INTERVENTION MIGHT WORK

#### 1.3.1 Theory of change

RHSs and CRCs seek to build upon the social connectedness contained within the educational environment (Finch & Frieden, 2014). The recovery school model draws heavily on the notion of social capital, or the social support, connections, and access to resources available through social networks, as one of the strongest factors influencing educational and recovery outcomes (Coleman, 1988; Cloud & Granfield, 2004; Meier, 1999). Connectedness between youth, family, peers, school, and community generates social capital and can potentially translate into academic achievement for youth (Meier, 1999).

RHSs support the recovery and academic achievement of students by fostering connectedness and social capital in a setting that addresses issues related to addiction and recovery with dedicated attention to healthy adolescent growth and development. They provide a safe environment for youth in recovery to practice the skills learned in treatment, use structured activities so that youth learn how to engage with their own emotional state and physiological responses they previously avoided through substance use, and use the sober community to model positive behaviors of peers and mentors (Finch & Frieden, 2014). The primary elements through which this underlying theory of change is operationalized are: build a richer base of peer and family connection, social support, and accountability; minimize contact with negative peers to increase school engagement and reduce the risk of returning to use; provide students the opportunity to meet peers in recovery and to incorporate skills learned in treatment; quickly respond to problematic behaviors or symptoms of a co‐occurring disorder due to the small school environment and specialized school staff; promote contact with positive peers and adults outside school by requiring participation in support groups after school; and support graduation from high school by providing an accredited curriculum taught by licensed teachers (Tanner‐Smith et al., 2014).

Similar to RHSs, CRCs also support students' academic goals through fostering a positive, sober peer environment that provides companionship as well as emotional and recovery social support (Harris et al., 2014). Youth in recovery who choose to enroll in postsecondary education have additional tasks that can challenge their recovery. For example, unlike youth attending high school, youth in college are tasked with finding their own network of social support and developing healthy peer relationships, moving away from home and learning to live outside of their caregiver's supervision, and setting and attaining educational and career goals (Harris et al., 2008). For youth in recovery, these tasks must be undertaken with the added pressure of finding a peer environment that supports abstinence or sobriety, a difficult task in an abstinence‐hostile environment. Thus, through their available programming and dedicated on‐campus recovery support, CRCs seek to create an internal community of recovery support, advocate for sobriety among the larger community, and support students as they transition to young adulthood. CRCs are vital environmental supports for youth in recovery wishing to attend postsecondary education: indeed, in a recent survey of students in 29 CRCs across the United States, one‐third of respondents stated they would not have chosen to attend higher education without a campus recovery support system (Laudet et al., 2016).

Although RHSs and CRCs operate along a similar theory of change in relation to sober peer support and community, they have different operating models and structures based on how they seek to intervene with youth and due to their placement in particular educational systems. For example, RHSs operate as both the formal school and recovery support system and there may be less of a clearer distinction between the classroom as an academic versus recovery space. As part of the educational system, RHSs are also able to award their students secondary diplomas when they graduate. Alternatively, CRCs are operated on a college campus but are primarily separate from academic classroom spaces, offering additional supports outside of the academic structure. Youth in CRCs may choose to discontinue their participation in the CRC activities but still remain enrolled at the college, while youth at RHSs need to remain sober for continued enrollment at the school. When RHS youth return to use substances, these situations are typically treated on a case‐by‐case basis, but if the youth continues to use substances without indicating motivation to change, they are eventually referred to another school setting to preserve the sober environment of the RHS. Given the close proximity of students and teachers and small school size of RHSs, there is also closer monitoring of youth behavior at the RHS, compared to CRCs, which are housed on larger campuses.

#### 1.3.2 Factors that affect youth experience of recovery schools

Multiple, interacting factors may affect whether participants enroll in or join a recovery school and once participating, will determine how the recovery school setting affects subsequent substance use and other behavioral outcomes. Most often studied are individual‐level factors such as demographic and socioeconomic characteristics.

Sex/gender and age have distinguished different pathways of youth recovery involvement and outcomes (Stevens, Estrada, Murphy, McKnight, & Tims, 2004; Wellman, Contreras, Dugas, O'Loughlin, & O'Loughlin, 2014). For example, male college students have higher annual prevalence rates of use of marijuana and most other drugs than female college students (Dennhardt & Murphy, 2013). There are also differences between males and females on mental health comorbidity and level of juvenile justice involvement (Stevens et al., 2004), which may interact with level of substance use and/or level of care received for that use (Oser, Karakos, & Hennessy, 2016). Mental health, specifically diagnoses of mental illness comorbidities, has also been studied in this population and found important to outcomes (Hersh, Curry, & Yaminer, 2014; Yu, Buka, Fitzmaurice, & McCormick, 2006). For example, one review of comorbid depression and adolescent treatment outcomes indicated mixed results depending on study characteristics and concluded that there is not a simple way to categorize the relationship between depression and substance use outcomes (Hersh et al., 2014).

Finally, evidence suggests that tangible resources affect recovery, possibly through either restricting or enhancing access to supportive social networks and other established recovery supports (Granfield & Cloud, 1999; Pesetski, 2015; Terrion, 2012); thus, measures of socioeconomic status are important indicators of potential inequity among this population.

### 1.4 WHY IT IS IMPORTANT TO DO THE REVIEW

To our knowledge, no reviews of CRCs exist in the literature. Further, there is only one systematic review to date that has included findings from RHSs (Fisher, 2014). That systematic review identified a range of adolescent‐specific continuing care supports, however, and did not solely focus on RHSs. In addition, although four studies of RHSs were identified in the review, the author had limited resources with which to conduct the review and a meta‐analysis was not attempted.

Other reviews on adolescent treatment and recovery have compared the effectiveness of different adolescent outpatient treatments (Tanner‐Smith et al., 2013; Waldron & Turner, 2008), reviewed adolescent participation in 12‐Step programs (Kelly & Myers, 2007; Sussman, 2010) and the role of social support in collegiate recovery programs (Smock, Baker, Harris, & D'Sauza, 2011), and explored the relationship between 12‐Step attendance and adolescent substance use outcomes posttreatment (Hennessy & Fisher, 2015). Although these reviews have highlighted the importance of treatment and support programs for youth with SUDs, they have not focused explicitly on examining the potential effectiveness of recovery schools. Thus, there is a need to understand the effects of education‐based interventions in light of our understanding of other youth recovery supports especially given the recent enthusiasm for their implementation (NIDA, 2014; ONDCP, 2014; White, 2009) and recovery school funding mechanisms in some U.S. states (Finch et al., 2016).

Finally, based on resources located from U.S.‐based national networks and recent documents outlining the recovery school movement (ARHE, 2016; Laudet et al., 2014; White & Finch, 2006), we anticipated that the majority of recovery school programs would be located in the United States. We are aware of at least one potential recovery school program in China (Lin, Lu, & Wu, 2017). However, we are not aware of other attempts to locate recovery school program literature on an international scale. An extensive systematic search enables knowledge around whether, and if so, where such programs are in operation worldwide. Substance use disorders among youth are problematic in every nation, and many countries have instituted treatment and recovery resources specific to adolescents and young adults. Thus, this review aims to highlight gaps in school‐focused recovery supports and potential solutions for addressing youth recovery moving forward.

## 2 Objectives

Recovery school programs in the United States are now operating with a substantial infrastructure and both private and government funding are made available for these supports (Finch et al., 2016; Moberg & Finch, 2008; Oser et al., 2016). Globally, there is also increased attention to building more comprehensive, developmentally‐appropriate continuing care supports to break the cycle of relapse and return to treatment that many youth experience (Daddow & Broome, 2010; Sussman, 2010; White, Kelly, & Roth, 2012). Thus, this review summarized and synthesized the available research evidence on the effects of recovery schools for improving academic success and social and emotional well‐being among high school and college students who are in recovery from substance use. The specific research questions that guided the review are as follows:

1. What effect does recovery school attendance (versus attending a non‐recovery or traditional school setting) have on academic outcomes for students in recovery from substance use? Specifically (by program type):
  a. For recovery high schools: what are the effects on measures of academic achievement, high school completion, and college enrolment?
  b. For collegiate recovery communities: what are the effects on measures of academic achievement and college completion?
2. What effect does recovery school attendance have on substance use outcomes for students in recovery from substance use? Specifically, what are the effects on alcohol, marijuana, cocaine, or other substance use?
3. Do the effects of recovery schools on students' outcomes vary according to the race/ethnicity, gender, or socioeconomic status of the students?
4. Do the effects of recovery schools on students' outcomes vary according to existing mental health comorbidity status or juvenile justice involvement of the students?

## 3 Methods

The methods for this systematic review are based on the protocol published in the Campbell Collaboration Library of Systematic Reviews (Hennessy, Tanner‐Smith, Finch, Sathe, & Potter, 2017). The protocol can be accessed at https://www.campbellcollaboration.org/library/recovery‐schools‐students‐in‐recovery‐from‐substance‐use.html

### 3.1 CRITERIA FOR CONSIDERING STUDIES FOR THIS REVIEW

#### 3.1.1 Types of studies

This review included studies that used an experimental or quasi‐experimental design. For the primary analysis, eligible studies were those that compared outcomes for students enrolled in recovery school programs with students enrolled in one or more comparison conditions that did not involve a recovery school program. To be considered eligible, study designs were required to meet one of the following criteria:

i. Randomized controlled trial (RCT): Participants were randomly assigned to intervention and comparison conditions. Individual and cluster level randomization was acceptable.
ii. Quasi‐randomized controlled trial (QRCT): Participants were assigned to intervention and comparison conditions via a quasi‐random procedure, such as birth date or student record number.
iii. Quasi‐experimental controlled trial (QED) with individual level matching: Participants in the intervention and comparison conditions were allocated to conditions via a non‐random process, but participants were individually matched on at least one measure of substance use and on three student demographic characteristics (age, race/ethnicity, gender).
iv. Quasi‐experimental controlled trial (QED) with pretest‐adjusted outcomes: Participants in the intervention and comparison condition were formed via a non‐random process, but the study authors adjusted for pretest differences between groups (e.g., as pretest‐adjusted posttest means, regression coefficients from models that adjusted for pretest). For those outcomes on which pretest data were not applicable (e.g., high school graduation), adjustment must have been done for a close proxy of a pretest.
v. Quasi‐experimental controlled trial (QED) with pretest data: Participants in the intervention and comparison condition were formed via a non‐random process, but pretest data were available for each outcome. Pretest data must have been reported in a form that permitted assessment of the initial equivalence of the intervention and control groups on those variables via calculation of an effect size. For those outcomes on which pretest data were not applicable (e.g., high school graduation), data for a close proxy of a pretest must have been available.

#### 3.1.2 Types of participants

Eligible student populations included students enrolled part‐time or full‐time in secondary (high school) or postsecondary (college or university) educational institutions. All ages of students were eligible for inclusion provided they were enrolled in an educational institution at the time of the study, but most secondary and postsecondary students were expected to be ages 14–25. Studies that included students who were not enrolled in educational institutions at the time of the intervention were not eligible for inclusion.

Eligible student populations were those comprised of students in recovery from substance use. The definition for “in recovery” is broad, and encompasses any student with a history of using substances who is motivated or interested in reducing their substance use or maintaining abstinence from substance use. Students who were mandated to a program because of behaviours related to substance use or actual problematic use were also considered eligible populations. Due to inconsistent reporting in primary studies, students in recovery were not required to have a formal SUD diagnosis (e.g., substance abuse or substance dependence diagnosis based on DSM criteria).

To be as inclusive as possible, no other eligibility restrictions were placed on the eligible participant populations. Students in recovery who were enrolled in educational institutions in any country were eligible for inclusion.

#### 3.1.3 Types of interventions

The review included any recovery school program that was designed to support the academic success of students in recovery from substance use. Recovery schools are broadly defined as educational institutions, or programs at educational institutions, that are developed specifically for students in recovery and address recovery needs in addition to academic development. Eligible recovery school programs could be at the secondary (Recovery High Schools [RHS]) or postsecondary level (College Recovery Communities [CRC]) and should meet the standards detailed by the Association of Recovery Schools (2013) or by the Association of Recovery in Higher Education (2015) as outlined below. Both sets of guidelines provided by the two organizations are broad enough to include schools and programs of varying designs, yet stringent enough to exclude treatment‐based programs to ensure the review's focus on academic recovery supports. Although RHSs were required to meet the broad standards detailed by the ARS to be considered eligible for the review, these programs did not need to be officially accredited by ARS to be included in the review (because the accreditation process is fairly new, may be costly for some locations, has not been widely implemented at this time, and is currently only available in the United States) (ARS, 2016). Given the different models of RHSs and CRCs, eligibility criteria differed by program type.

*Recovery High Schools (RHSs)* were required to meet the inclusion criteria described below to be eligible for inclusion in the review. Eligible RHS programs must have:

i. had as their primary mission to provide education to youth in recovery from substance use or co‐occurring disorders;
ii. had an explicit goal of providing academic or educational instruction to high school students;
iii. had an explicit goal of providing a high school environment oriented around recovery from substance use or co‐occurring disorders and require previous treatment and/or commitment to sobriety;
iv. met state requirements for awarding a secondary diploma;
v. had direct contact with one or more students, and provided educational instruction via face‐to‐face, online, telephone, or video communication;
vi. been available to any student in recovery from substance use or co‐occurring disorders who met state or district eligibility requirements for attendance.

*Collegiate Recovery Communities (CRCs)* must have met the inclusion criteria described below to be eligible for inclusion in the review. Eligible CRCs must have:

i. had an explicit goal of providing recovery support services and focused on encouraging abstinence from use of substances;
ii. had an explicit goal of providing a collegiate environment oriented around recovery from substance use, which could be met through any of the following or a combination of the following activities for youth in recovery: (a) had a dedicated space for students in recovery to meet and support each other, (b) offered group mutual aid meetings, and/or (c) offered individual counseling services with trained, specialized staff;
iii. been located in a postsecondary educational setting, had direct contact with one or more students, and provided supports via face‐to‐face, online, telephone, or video communication.

*Comparison Condition.* Eligible comparison conditions were those that included traditional educational programs or services that did not explicitly have a substance use recovery focus. A school specifically designed for students with mental health diagnoses who did not have a substance use disorder was also considered to be an eligible comparison condition.

#### 3.1.4 Types of outcome measures

##### 3.1.4.1 Primary outcomes

The primary outcomes eligible for this review are divided into two broad domains, with further subdivisions for constructs within each of these domains. Studies that met all other eligibility criteria were considered eligible for the narrative review portion of this review even if they did not report outcomes in one of the primary outcome domains.

*Academic performance domain*. The academic performance domain includes outcomes that assess students' academic achievement and performance in school. Eligible constructs within this domain include standardized achievement test scores (e.g., ACT, SAT, state assessments), grade‐point average (GPA), high school completion or attendance, college enrolment, and college completion. College enrolment and completion outcomes can be at any type of postsecondary educational establishment including two‐ and four‐year institutions and technical colleges. However, given differences in credit value across institutions, the number of credits earned toward a high school or college diploma were not considered eligible outcomes.

*Substance use domain*. The substance use domain includes outcomes that assess students' consumption of alcohol and other illicit substances. Eligible constructs in this domain included alcohol, marijuana, cocaine, heroin, stimulant, and other substance use (i.e., general measures that collapse across different types of substances). Tobacco use (and its respective DSM diagnoses) and caffeine use were not eligible constructs in this domain. Official DSM diagnoses including an abuse, dependence, or substance use disorder diagnosis, were eligible constructs within this domain. Diagnostic outcomes were not limited to particular scales as long as the scale used was a standardized and/or validated measure.

##### 3.1.4.2 Secondary outcomes

There is one domain of secondary outcomes eligible for this review: substance use related problems. This domain included measures of problems related to the consumption of alcohol and/or substances, such as arrests, DUI/DWI (e.g., motor vehicle problems), health consequences, peer and family relationship problems, risky sexual behaviour, school or work problems, and mixed negative consequences as measured by a scale (e.g., Rutgers Alcohol Problem Index [RAPI] score; White & Labouvie, 1989). Studies that met all other eligibility criteria were eligible for the review even if they did not measure a secondary outcome.

#### 3.1.5 Duration of follow‐up

There were no eligibility criteria based on duration of follow‐up. All follow‐up periods of eligible studies were considered.

#### 3.1.6 Types of settings

Eligible studies could be conducted in any country. Eligible settings for intervention delivery were educational settings for youth in recovery and are described in detail in the section discussing intervention eligibility criteria (above). If the location of the educational program was in a substance use treatment center, then the program was not eligible for inclusion in this review because educational programs delivered in formal substance use treatment settings prioritize treatment services over academics and may involve more intensive treatment services. Thus, programs in these settings did not qualify as a posttreatment or continuing care recovery support environment.

#### 3.1.7 Other eligibility criteria

Eligible studies could have been published in any language and reported in any form or type of publication, including but not limited to journal articles, books, book chapters, theses and dissertations, technical reports, conference papers, and other unpublished but disseminated formats. Studies were required to have been reported in 1978 or later, given that the first collegiate recovery program was developed in 1977 (White & Finch, 2006) and the first recovery high school was developed in 1979 (A. Finch, personal communication, June, 15, 2015; Ruben, 2000).

### 3.2 SEARCH METHODS FOR IDENTIFICATION OF STUDIES

We conducted extensive searches of electronic databases, hand searches of journals, and grey literature sources to identify all potentially eligible published and unpublished studies.

#### 3.2.1 Electronic searches

As addiction and recovery supports such as recovery schools span multiple disciplines, education, social science, and public health electronic databases were searched. Search terms varied by database, but generally included three blocks of terms and appropriate Boolean or proximity operators, if allowed: blocks included terms that addressed (1) intervention; (2) population; (3) outcomes. We searched the following 11 electronic databases (hosts): Cochrane Central Register of Controlled Trials (CENTRAL), Cochrane Database of Abstracts of Reviews of Effects (DARE), Education Resources Information Center (ERIC, via ProQuest), Education Database (via ProQuest), International Bibliography of the Social Sciences (IBSS, via ProQuest), PsycINFO (via ProQuest), PsycARTICLES (via ProQuest), PubMed, Social Services Abstracts (via ProQuest), Sociological Abstracts (via ProQuest), and Web of Science. See Table 1 for the full search strings used in the ProQuest, PubMed, and Web of Science hosts. All electronic searches were originally completed in November of 2016 and an update was completed July 29, 2018.

**Table 1:** Search strings for electronic databases

**Databases (Host)**	**Search Terms**
ERIC, IBSS, ProQuest Education Database, ProQuest Dissertations & Theses Global, PsycARTICLES, PsycINFO, Social Services Abstracts, Sociological Abstracts (ProQuest)	(TI,AB(“recovery high school” OR RHS OR “recovery school” OR “sober school” OR “sober high school” OR “collegiate recovery communities” OR “collegiate recovery community” OR CRC OR “collegiate recovery program*” OR CRP OR “college recovery community” OR “college recovery program” OR “campus recovery program” OR “campus recovery community” OR “recovery community” OR (school NEAR recovery))) AND (alcohol OR drink* OR substance OR drug OR marijuana OR cannabis OR cocaine OR amphetamine OR heroin OR inhalant OR opioid OR opiate OR “substance use disorder” OR “substance abuse” OR “drug abuse” OR addiction) AND (student* OR youth OR adolescen* OR teen OR teens OR teenager OR teenagers OR “young adult” OR “young adults” OR “emerging adult” OR undergraduate OR undergraduates)
*PubMed*	((substance abuse treatment centers [mh] AND schools [mh]) OR (“recovery school” [tiab] OR “recovery high school” [tiab] OR “recovery high schools” [tiab] OR “alternative school” [tiab] OR “alternative high school” [tiab] OR “college recovery community” [tiab] OR “campus recovery program” [tiab] OR “campus recovery community” [tiab] OR “recovery community” [tiab])) AND (drinking behavior [mh] OR adolescent behavior [mh] OR drug‐seeking behavior [mh] OR health behavior [mh] OR marijuana smoking [mh] OR substance‐related disorders [mh] OR “substance abuse” [tiab] OR “drug abuse” [tiab] OR drinking [tiab] OR alcohol [tiab] OR drugs [tiab] OR marijuana [tiab] OR cannabis [tiab] OR cocaine [tiab] OR amphetamine [tiab] OR heroin [tiab] OR inhalant [tiab] OR opioid [tiab] OR opiate [tiab] OR addict* [tiab])
*Web of Science*	(TS=(recovery high school OR RHS OR “recovery school” OR sober school OR sober high school OR “collegiate recovery communities” OR collegiate recovery community OR “collegiate recovery program” OR college recovery community OR college recovery program OR campus recovery program OR campus recovery community OR recovery community OR (school NEAR recovery))) AND (TS=(alcohol OR drink OR substance OR drug OR marijuana OR cannabis OR cocaine OR amphetamine OR heroin OR inhalant OR opioid OR opiate OR substance use disorder OR substance abuse OR drug abuse OR addiction)) AND (TS=(student OR youth OR adolescent OR teen OR teens OR teenager OR teenagers OR young adult OR young adults OR emerging adult OR undergraduate OR undergraduates))

#### 3.2.2 Searching other resources

As suggested in the Campbell Systematic Review literature search guide (Kugley et al., 2016), we originally planned to conduct journal hand searches in the most current issues of journals where a large number of potentially eligible studies were found. However, given that only one unpublished manuscript was ultimately eligible for inclusion in the review, we instead conducted hand searches of journals in which at least five retrieved references were located. Thus, we conducted hand searches for the following 17 journals: *Addiction*, *Addictive Behaviors*, *Alcoholism Treatment Quarterly*, *Archives of Disease in Childhood*, *Child Welfare*, *Drug and Alcohol Dependence*, *Health Psychology*, *Journal of Cancer Education*, *Journal of Child & Adolescent Substance Abuse*, *Journal of Drug Education*, *Journal of Groups in Addiction & Recovery*, *Journal of Psychoactive Drugs*, *Journal of School Health*, *Journal of Social Work Practice in the Addictions*, *Journal of Substance Abuse Treatment*, *Peabody Journal of Education*, and *The American Journal on Drug & Alcohol Abuse*. All hand searches were originally completed in February of 2017 and were updated on September 12, 2018.

The following 11 websites were also searched for grey literature sources: Alcohol Studies Database (http://www2.scc.rutgers.edu/alcohol_studies/alcohol/), Association of Recovery in Higher Education (http://collegiaterecovery.org), Association of Recovery Schools (https://recoveryschools.org), Drug & Alcohol Findings Project (http://findings.org.uk/index.php?s=eb), International Clinical Trials Registry (http://apps.who.int/trialsearch/), National Institute on Alcohol Abuse and Alcoholism (https://www.niaaa.nih.gov), NIH RePORTER (https://projectreporter.nih.gov/reporter.cfm), ProQuest Dissertations and Theses Global (https://search.proquest.com/pqdtglobal/), SafetyLit (http://www.safetylit.org/index.htm), SAMHSA (http://www.samhsa.gov), and Theses Canada (http://www.bac‐lac.gc.ca/eng/services/theses/Pages/theses‐canada.aspx). All website searches were originally completed in October of 2016 and were updated on July 29, 2018.

Leading authors and experts in the field of youth addiction and recovery were also contacted for additional studies via email in February of 2017. The bibliographies of relevant reviews and included studies were also searched to identify additional references for review. Finally, in February of 2017 and then again in September 2018 we conducted forward citation searching using the website Google Scholar. We chose Google Scholar for forward searching since this database produces similar results to other search engines such as Web of Science (Tanner‐Smith & Polanin, 2015).

### 3.3 DATA COLLECTION AND ANALYSIS

#### 3.3.1 Selection of studies

Two reviewers independently screened studies' titles and abstracts to assess for potential eligibility; any disagreements were resolved via discussion and consensus. Potentially eligible studies were then retrieved in full text and these full texts were reviewed for eligibility, again using two independent reviewers. Disagreements between reviewers were resolved via discussion and consensus. If eligibility could not be determined due to missing information in a report, we contacted study authors for this information. We used FileMaker Pro for the entire literature screening and data extraction process.

#### 3.3.2 Data extraction and management

Two graduate‐level student reviewers were trained on the coding manual by the review's primary author (EAH). All studies were independently double‐coded by these two reviewers, using a predetermined codebook (see Hennessy et al., 2017 for the codebook). Coding disagreements between the two reviewers were resolved via discussion and consensus. Because the sole study eligible for the review was conducted by some of the authors of this review (AJF, EAH, ETS), this study was coded by the two graduate student reviewers who were not connected to the study. This process, including discrepancy resolution, was supervised by a senior reviewer not connected to the study (NS).

The primary categories for coding were as follows: participant demographics (e.g., age, DSM diagnoses for mental health and substance use, gender, grade level, race/ethnicity, socioeconomic status); intervention setting (e.g., country, academic level of focus, urbanicity); intervention components and curriculum delivery (e.g., community service, parent involvement, online coursework, in‐person lessons); study characteristics (e.g., attrition, duration of follow‐up, study design, participant dose, sample N); outcome construct (e.g., type, description of measure); outcome results (e.g., timing at measurement, baseline and follow‐up mean and standard deviation). Additionally, external peer reviewers requested that additional data be extracted from the reports connected to the single eligible study. These additional data included study aims, manuscript focus, timing of report, sample size at the time of the report, type of data collected/presented, and outcomes reported and were collected from those ancillary reports by EAH and checked by NS.

#### 3.3.3 Assessment of risk of bias in included studies

The risk of bias in included studies was assessed using the Cochrane risk of bias tool for

non‐randomized studies (Sterne et al., 2016), the ROBINS‐I. This tool requires that at the protocol stage of the review, two sets of items are determined: (1) confounding factors and (2) co‐interventions that could be different between groups with the potential to differentially impact outcomes. For this review, we specified the following confounding factors at the protocol stage: prior substance use, prior academic achievement, mental health comorbidities, and readiness to change. We also specified the following co‐interventions as potentially different between groups: self‐help attendance (e.g., 12‐Step programs) and other substance use counseling services (e.g., outpatient treatment).

We used the ROBINS‐I tool to assess risk of bias for all seven risk of bias subdomains and an assessment of the overall risk of bias for the study. Because the sole study eligible for the review was conducted by some of the authors of this review (AJF, EAH, ETS), two graduate‐level reviewers extracted data using the ROBINS‐I tool, and a senior reviewer (NS) resolved any discrepancies.

If we had identified any eligible randomized studies, we would have used the Cochrane risk of bias tool for randomized studies (Higgins & Green, 2011); however, no eligible randomized studies were identified in our search.

#### 3.3.4 Measures of treatment effect

We anticipated that outcomes in included studies would likely be reported as continuous measures using a variety of scales (e.g., numeric grades or test scores, frequency or quantity of use); thus, the standardized mean difference (SMD; *d*) was the primary effect size metric used to quantify study findings. All standardized mean difference effect sizes were corrected with the small sample adjustment (Hedges' *g*). Effect sizes were coded such that positive values (> 0) indicated a beneficial effects of recovery schools (i.e., higher academic achievement, less substance use).

For binary outcomes (e.g., abstinence from drugs), we used the odds ratio effect size metric to quantify study findings. We assumed that binary outcomes reflected different underlying constructs than continuous measures (i.e., abstinence from substance use represents a different underlying construct than frequency or quantity of substance use); therefore, all analyses presented the Hedges' *g* and odds ratio effect sizes separately.

To minimize any potential bias in the meta‐analysis results introduced by effect size outliers, we planned to Winsorize any effect sizes that were three or more standard deviations from the mean (Lipsey & Wilson, 2001); however, because only one study met eligibility criteria and was included in the review, we did not Winsorize any effect sizes as planned.

#### 3.3.5 Unit of analysis issues

All reports of unique studies were reviewed to ensure that articles reporting on the same study were appropriately linked so that only unique study samples were included in each analysis. In our protocol we had originally planned to deal with within‐study effect size dependency based on how many studies were eligible for the review, because some meta‐analytic methods for handling statistical dependencies (e.g., robust variance estimation) require large numbers of included studies (e.g., see Tanner‐Smith & Tipton, 2014 for a summary). However, there was only one eligible study so no meta‐analyses were conducted.

#### 3.3.6 Dealing with missing data

We planned to contact primary study authors if data needed to calculate an effect size were missing from a report. In the one eligible study included in the review, no additional data were needed or requested from the authors.

#### 3.3.6 Assessment of heterogeneity

We originally planned to assess and report heterogeneity using the *x* ^2^ statistic and its corresponding *p* value, *I* ^2^, and *τ* ^2^, but because only one eligible study was identified, a meta‐analysis was not possible and the assessment of effect size heterogeneity was not applicable.

#### 3.3.7 Assessment of reporting biases

We planned to use assessments appropriate for reporting and publication biases, such as the contour enhanced funnel plot (Palmer, Peters, Sutton, & Moreno, 2008), regression test for funnel plot asymmetry (Egger, Smith, Schneider, & Minder, 1997), and the trim and fill method (Duval & Tweedie, 2000). However, because only one eligible study was identified and a meta‐analysis was not performed, we did not conduct any assessments of reporting biases.

#### 3.3.8 Data synthesis

Given the expected diversity of populations and settings, we originally planned to conduct meta‐analyses using random‐effects inverse variance weights and to report 95% confidence intervals, with mean effect sizes reported separately by study design (RCTs and non‐RCTs), intervention type (RHSs and CRCs), and outcome domain (academics, substance use, and substance use problems). Because only one eligible study was identified and a meta‐analysis was not performed, we instead narratively present effect size estimates and their respective 95% confidence intervals for each outcome domain.

#### 3.3.9 Subgroup analysis and investigation of heterogeneity

We planned to explore the following moderators using mixed‐effect meta‐regression models: (1) proportion of males; (2) mean age of sample; (3) proportion of students with mental health comorbidities; (4) proportion of students with juvenile justice involvement; (5) student socioeconomic status; (6) and student race/ethnicity. These effect size moderators have been established in prior research as potentially important factors in the role of youth recovery from substance use and were addressed in the background section of this review. Again, however, given that only one eligible study was identified and a meta‐analysis was not possible, we were unable to conduct the planned meta‐regression models. We instead narratively describe these characteristics for the one included study.

#### 3.3.10 Sensitivity analysis

We originally planned sensitivity analyses to assess the robustness of review findings which included: (1) level of within‐study attrition; (2) whether or not a standardized assessment tool was used to measure outcomes; and (3) whether removal of Winsorized effect sizes to handle potential outliers substantively altered the review findings. Again, however, we were unable to conduct these sensitivity analyses given that only one study was eligible for inclusion in the review.

### 3.4 DEVIATIONS FROM THE PROTOCOL

There were some deviations from the published protocol. After submission of the draft review, external peer reviewers requested additional data to be collected on the reports connected to the single eligible study. This additional information was not part of our original codebook and included the following: study aims, manuscript focus, timing of report, sample size at the time of the report, type of data collected/presented, and outcomes reported. Due to resource constraints, this information was not double‐coded, rather it was collected from those supplementary reports by EAH and checked by NS. However, none of the information collected was integral to the outcomes reported in this review as these reports were primarily focused on methods approaches for the eligible study and/or presentation of preliminary or descriptive outcomes (i.e., when recruitment was ongoing). Additionally, because only one study met the inclusion criteria for the review, we did not conduct any of the quantitative syntheses that were planned. As newly eligible studies become available, future updates to the review will include the quantitative syntheses (and corresponding heterogeneity analyses, moderator analyses, and publication bias analyses) as specified in the protocol.

## 4 Results

### 4.1 RESULTS OF SEARCH

**Figure 1:**
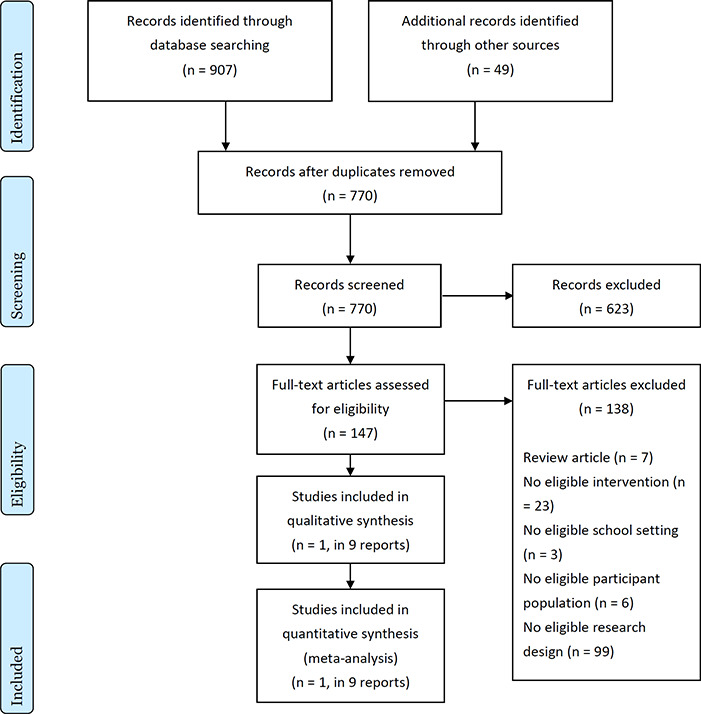
Flow diagram of studies

Figure 1 presents the flow diagram of studies identified in the literature search.

Original search: A total of 720 records were identified through electronic database searching and an additional 40 records were identified through our supplementary searches; 82 duplicates were removed, leaving 639 unique records that were screened for eligibility. At the abstract screening level, 507 of the 639 (79%) records were identified as clearly ineligible, and removed from further consideration. The remaining 132 records were retrieved and screened for eligibility at the full‐text level. Of the 132 records screened for eligibility, 125 were deemed ineligible: 6 were review articles, 21 did not include an eligible RHS or CRC intervention, 3 were studies conducted outside of school settings, 4 did not include eligible populations of students with substance use problems, and 91 did not use eligible research designs to evaluate the effects of a recovery school program. Only one study (described in nine reports) met our inclusion criteria and was included in the review.

Updated search conducted in 2018: A total of 187 records were identified through electronic database searching and an additional 9 records were identified through our supplementary searches; 65 duplicates were removed, leaving 131 unique records that were screened for eligibility. At the abstract screening level, 116 of the 131 (89%) records were identified as clearly ineligible, and removed from further consideration. The remaining 15 records were retrieved and screened for eligibility at the full‐text level. Of the 15 records screened for eligibility, 13 were deemed ineligible while two were linked to the sole included study: one was a review article, two did not include an eligible RHS or CRC intervention, two did not include eligible populations of students with substance use problems, and eight did not use eligible research designs to evaluate the effects of a recovery school program.

#### 4.1.1 Included studies

##### 4.1.1.1 Recovery high schools

Of the 956 total reports identified in the systematic literature search, only one study met the inclusion criteria for the review, which we describe in detail below (see Table 2). This study examined the effects of RHS attendance on academic and substance use outcomes among U.S. adolescents (Finch, Tanner‐Smith, Hennessy, & Moberg, 2018; supplementary reports from the same study Botzet, McIlvaine, Winters, Fahnhorst, & Dittel, 2014; Finch & Moberg, 2013; Finch, Moberg, Tanner‐Smith, & Hennessy, 2015; Hennessy, 2018; Moberg, Finch, & Lindsley, 2014; Tanner‐Smith, Finch, Hennessy, & Moberg, 2017; Tanner‐Smith, Finch, Hennessy, & Moberg, 2018; Tanner‐Smith & Lipsey, 2014). At the time of our original search, this study was described in three published journal articles, two unpublished manuscripts under review, and two conference presentations; however, after external peer review of our manuscript, the list of reports for this single study had grown to six published journal articles, one dissertation, and two conference presentations. The Finch and colleagues (2018) report, “Recovery high schools: Effect of schools supporting recovery from substance use disorders” was the manuscript from this study that reported the outcomes of interest for this review and was the primary source of information from which data were collected, although all manuscripts were reviewed to ensure the study was comprehensively coded. Three of the other published manuscripts described elements of the study design and preliminary descriptive outcomes (Botzet et al., 2014; Moberg et al., 2014; Tanner‐Smith & Lipsey, 2014) while one described mental health outcomes (Tanner‐Smith et al., 2018a). Another publication compared the study sample to a national sample of youth in SUD treatment across the United States using a de‐identified multisite data set managed by Chestnut Health Systems (Tanner‐Smith et al., 2018b). The dissertation was an exploratory secondary data analysis of the partial data set (Hennessy, 2017) while the two conference presentations described preliminary findings of the study while recruitment was ongoing (Finch & Moberg, 2013; Finch et al., 2015). Additional information about these supplementary reports can be found in Table 3.

**Table 2:** Characteristics of included studies

*Finch et al., 2018*	
**Study Features**	**Description**
*Design*	Controlled quasi‐experimental design (QED); students self‐selected into intervention (RHS) and comparison (non‐RHS) conditions. The matched analytic sample included 194 participants (*n_TX_* = 134; *n_CT_* = 60).
*Location*	Three U.S. states (MN, TX, and WI).
*Participants*	*Intervention group*: Mean age 16.49 (11^th^ grade), 85% White, 50% male, 58% with college‐educated parents, 91% with mental health treatment history. *Comparison group*: Mean age 16.25 (11^th^ grade), 87% White, 52% male, 58% with college‐educated parents, 86% with mental health treatment history.
*Intervention*	Students attended at least one recovery high school for at least 28 days during the study period.
*Outcomes*	Academic Performance Domain *Grade point average*: Grade point average in English/reading, mathematics, and other high school subjects in past 90 days (self‐report). *Truancy*: Recency of skipping school in the past 90 days (self‐report). *Absenteeism*: Days absent from school in the past 90 days (self‐report). Substance Use Domain *Alcohol use*: Days used alcohol in past 90 days (self‐report, using Timeline Followback method). *Marijuana use*: Days used marijuana in past 90 days (self‐report, using Timeline Followback method). *Other drug use*: Days used drugs other than alcohol or marijuana in past 90 days (self‐report, using Timeline Followback method). *Abstinence from alcohol/drugs*: Complete abstinence from alcohol, marijuana, or other drugs in past 90 days (self‐report, using Timeline Followback method).
*Follow‐up (months)*	6
*Attrition (%)*	22%

**Table 3:** Characteristics of supplemental reports for included studies

**Study**	**Bulleted aims**	**Manuscript Focus**	**Study N (****Finch et al., 2018**)	**Study Sample Type (** **Finch et al., 2018** **)**	**Other Sample Study N**	**Other Sample Type**	**Type of data collected**	**Student characteristics reported/ compared**	**Outcomes reported**
*Tanner‐Smith, & Lipsey, 2014*	1. Overview use of propensity scores in non‐randomized quasi‐experimental research 2. Describe importance of identifying/ measuring baseline covariates 3. Describe the approach used to identify covariates in the design of the recovery high school evaluation study	Analytic methods used in the study	NA	NA	NA	NA	NA	NA	NA
*Moberg et al., 2014*	1. Reviews findings from authors' earlier studies of recovery high schools 2. Presents early findings from a current study of the effectiveness of recovery high schools	Student characteristics	64	Students in 7 MN RHSs (2011–2013)	499	Students from Albuquerque RHS (1993–1995); Students from 17 RHS (2006–2009)	Interview, survey	Baseline characteristics (age, race/ethnicity, past substance abuse treatment type, past mental health treatment type, % depression, % juvenile justice involved, % substance use, tobacco use)	NA ‐ only report on differences in baseline characteristics between the samples
*Botzet et al., 2014*	1. Describe methodology used in the study of adolescents attending RHS or non‐RHS schools 2. Discuss key assessment strategies, recruitment, and interviewing methods used in the study 3. Provide recommendations for future research 4. Delineate developmental considerations inherent in research involving adolescents	Recruitment, assessment, and data collection methods used in the study	NA	NA	NA	NA	NA	NA	NA
*Tanner‐Smith et al., 2018a*	1. Examine the effects of RHS attendance on adolescents' mental health symptoms	Mental health outcomes	194 (RHS sample = 134, non‐RHS sample = 60)	All study sites (MN, TX, WI); matched participants	NA	NA	Standardized assessments	Baseline characteristics (age, school grade, % male, race, days used substances, received mental health treatment, age at first mental health treatment, ever received psychiatric medication, days of mental health services received)	Mental health symptoms (Any of the nine symptoms, major depressive disorder, generalized anxiety disorder, obsessive‐compulsive disorder, panic disorder, posttraumatic stress disorder, antisocial personality disorder, manic episode, hypomanic episode, suicide risk)
*Finch et al., 2015*	1. Describe RHS programs and need 2. Review preliminary outcomes 3. Review costs if need to be parallel with other studies	Preliminary student outcomes (presentation 2) and cost analysis (presentation 3)	108 (RHS sample=56, non‐RHS=52)	All study sites (MN, TX, WI); matched participants	NA	NA	Standardized assessments	NA	Presentation 2: Days of use (alcohol, marijuana, other drugs); School truancy, math grades, English grades. Presentation 3: Cost‐benefit analysis, pilot results
*Finch, & Moberg, 2013*	Description of study and preliminary outcomes (June 2013)	Preliminary student outcomes	250 recruited, but characteristics reported for baseline sample of 112 through May 2013. 3 ‐month unadjusted outcomes for 74 students (RHS=36, non‐RHS=38).	MN and WI sites (TX not yet a study site)	NA	NA	Standardized assessments	Baseline characteristics (age, sex, race/ethnicity, recruitment site, treatment history, substance use‐days alcohol, days marijuana, days other drugs, DSM‐IV diagnoses‐alcohol or drug abuse or dependence, tobacco dependent, depression)	3‐month outcomes (alcohol, marijuana, other drug use; depression problems, attending school, entered study from treatment center, whether other students “support my recovery”)
*Tanner‐Smith et al., 2018b*	1. To explore local differences in student characteristics among adolescents in recovery who select RHSs versus not 2. To explore macro‐level differences in adolescent characteristics of RHS students relative to a national sample of adolescents in SUD treatment	Characteristics of youth in recovery who elect to enroll in RHS	294 (RHS sample = 171, non‐RHS = 123)	All study sites (MN, TX, WI)	12,967	National sample of youth in treatment (GAIN)	Standardized assessments	Comparison of multiple characteristics: *Baseline characteristics*: age, sex, race/ethnicity, high school grade point average, any mental health diagnosis, any health insurance, private health insurance, single parent household, family history of substance use, family history of substance use treatment, family history of mental health problems, ever used alcohol to intoxication, ever used marijuana, ever used amphetamines, ever used cocaine/crack, ever opioids/narcotics, ever used hallucinogens, ever used PCP, ever used inhalants, days used alcohol (past 90), days used marijuana (past 90), days used other illicit drugs (past 90), tobacco dependence (past year), alcohol use disorder (past year), substance use disorder (past year), number of SUD treatment episodes, ever received residential SUD treatment, ever received intensive outpatient SUD treatment, ever received outpatient SUD treatment, ever received mental health treatment. *Adjustment characteristics after treatment discharge*: Spiritual Social Support Index, General Social Support Index, days attended/absent from school, days worked/absent from work, times in emergency room, days bothered by health problems, days health affected responsibilities, days in hospital, days on prescribed medication, times saw outpatient providers, times had outpatient surgery, days of illegal activity, days of illegal activity to get drugs, days of intoxicated illegal activity, times arrested, days on probation, days on parole, days in juvenile detention, days in jail/prison, days on house arrest, days on electronic monitoring, times had unprotected sex)	
*Hennessy, 2017*	1. Explore predictors of RHS versus non‐RHS attendance (paper 2) 2. Use exploratory methods to determine best covariates for use in propensity score estimation to balance RHS and non‐RHS groups (paper 3)	Factors leading to RHS attendance versus not (paper 2) Covariate selection for propensity scores (paper 3)	260 (RHS=120, non‐RHS=140)	All study sites (MN, TX, WI); January 2017, complete 6 month data	NA	NA	Standardized assessments	Age, age first treated for AOD, race/ ethnicity, sex, days of alcohol use/ marijuana use/other drug use, SUD diagnosis – alcohol/ other drugs, MH service receipt, AOD treatment, substance use expectancies – psychological and social benefits, negative attitudes towards school, general satisfaction, physical health, days of school attendance, cumulative GPA, stress, school problems, lifetime crime and violence, problem solving skill and orientation, DSM‐IV diagnoses, eating disorder screen, family income level, parental social position score, ever homeless, any health insurance, General Social Support Index, Social Competence Index, Neighborhood Social Connections Index, Spiritual Social Support Index, Substance approving peer attitudes, immediate family AOD/MH history, perceived drug availability knowledge of RHS prior to treatment, AOD/MH counseling outside school, AA/NA/12‐Step meeting attendance	Predictors of RHS attendance (paper 2); Covariates selected for propensity score estimation (paper 3); Days of marijuana use, days of alcohol use (paper 3)

The authors used an observational QED to examine the effects of RHSs on student outcomes. The study was conducted in the United States from 2011–2017. The study enrolled 293 adolescents and their caregiver(s) in three U.S. states (Minnesota, Wisconsin, and Texas) and followed them longitudinally over a 12‐month period. At the six month follow‐up period, the authors compared outcomes for students who self‐selected to attend RHSs relative to a comparison group of students who did not enroll in RHSs. Students enrolled in multiple types of schools during the study period, but the exact number of schools attended by students in the sample (RHS or otherwise) was not reported. The authors did not report the average duration (or minimum/maximum) number of days that the RHS students had been attending the RHSs; however, the authors defined the RHS intervention group as students who had enrolled in an RHS for at least 28 days during the study period. The authors did not report any other school‐level data regarding the RHSs represented in the sample (number of total students, number of teachers and staff, exact location of school, administrative structure).

***Intervention group characteristics.*** Youth enrolled in the RHSs were primarily White (85%; 7% were African American and 7% were an “other” race) and 50% were male. On average students were 16.49 years old (range: 14–19 years old). The breakdown by secondary school grade across the sample (9^th^/freshman, 10^th^/sophomore, 11^th^/junior, 12^th^/senior) was not provided, but students were on average in the 11^th^ grade (*Mean* = 11.09; *SD* = 0.91). In addition, 58% of the sample reported parental education greater than a bachelor‐level degree and 19% of the youth had some juvenile justice involvement. Mental health comorbidities among the sample of RHS students were high: 82% met criteria for major depressive disorder, 65% generalized anxiety disorder, 13% obsessive‐compulsive disorder, 36% panic disorder, 36% posttraumatic stress disorder, and 49% antisocial personality disorder. Almost all of the RHS students (91%) had received some form of mental health treatment service, 88% had ever been prescribed psychiatric medication, and at baseline reported receiving mental health services an average of 50 of the past 90 days (*SD* = 58.47).

***Comparison group characteristics.*** The comparison group of youth enrolled in non‐recovery high schools (i.e., a non‐recovery oriented school) were also primarily White (87%; 7% were African American and 7% were an “other” race) and 52% were male. On average students were 16.25 years old (range: 14–19 years old) and in the 11^th^ grade (*Mean* = 11.00; *SD* = 0.96). Mental health comorbidities among the comparison group students were also high: 75% met criteria for major depressive disorder, 63% generalized anxiety disorder, 12% obsessive‐compulsive disorder, 43% panic disorder, 22% posttraumatic stress disorder, and 35% antisocial personality disorder. Almost all of the comparison group students (86%) had received some form of mental health treatment service, 87% had ever been prescribed psychiatric medication, and at baseline reported receiving mental health services an average of 50 of the past 90 days (*SD* = 60.69). Of the students in the non‐RHS comparison group, “47% were attending traditional public high schools, 23% were attending non‐traditional high schools (including charter and alternative schools), 12% were attending online school, 5% were attending a technical school, and 8% were not enrolled in school” (Finch et al., 2018, p. 179).

***Primary study design and analysis.*** The authors used a longitudinal QED to compare outcomes for RHS students (intervention) vs. non‐RHS students (comparison). The authors used propensity scores to balance the intervention and comparison groups on the following baseline measures: age, race, gender, comorbid mental health conditions, alcohol use, tobacco use, other substance use, mental health treatment service receipt, physical health treatment receipt, life satisfaction, sources of life stress, criminal justice system involvement, perceived consequences of drug use, interest in attending an RHS, perceived teacher support for substance use recovery, prior year school attendance and grades, negative attitudes toward school, perceived academic abilities, school problems, family income, parental education level, parental history of substance use treatment, and family history of mental health problems (Finch et al., 2018, p. 177–178.). Students with propensity scores outside the region of common support (*n* = 35) were dropped from the outcome analysis; the final analytic sample size included 194 students (134 intervention, 60 comparison). To examine intervention effects, the authors used multilevel generalized linear regression models that controlled for the estimated propensity score and pretest measures of the outcomes.

##### 4.1.1.2 Collegiate recovery communities

Of the 760 total reports identified in the systematic literature search, no studies examining CRCs met the inclusion criteria for the review.

#### 4.1.2 Excluded studies

Of the 147 reports screened for eligibility at the full‐text level, 138 did not meet the inclusion criteria: 7 were review articles, 23 did not include an eligible RHS or CRC intervention, 3 were conducted outside of school settings, 6 did not include eligible populations of students with substance use problems, and 99 did not use eligible research designs to evaluate the effects of a recovery school program (see Table 4). Indeed, most identified reports that discussed RHSs or CRCs did not use quantitative comparison group designs to examine impacts; most articles simply described recovery schools in operation but did not include research or evaluation components. Among the few ineligible reports that did conduct research on recovery schools, all included pre‐experimental or observational research designs that lacked a comparison group.

**Table 4:** Exclusion characteristics

One hundred thirty‐eight studies were screened as ineligible for inclusion at the full‐text level; reasons for exclusion are listed below.	
**Study**	**Reason for Exclusion**
Addington (2015)	No eligible research design
ARS (2013)	No eligible research design
ARS (2016)	No eligible research design
Ashford (2018)	No eligible research design
Baker (2010)	No eligible research design
Bassuk (2016)	Review article
Beaty (2015)	No eligible research design
Beauvais (1986)	No eligible research design
Beeson (2017)	No eligible research design
Bell (2009a)	No eligible research design
Bell (2009b)	No eligible research design
Botzet (2008)	No eligible research design
Bourgeois (2008)	No eligible research design
Bowermaster (2008)	No eligible research design
Brown (2016)	No eligible research design
Browon (2018)	Review article
Bugbee (2016)	No eligible research design
Carothers (2006)	No eligible intervention
Casiraghi (2012)	No eligible research design
Casiraghi (2010)	No eligible research design
Castedo (2017)	No eligible research design
Clark (2010)	No eligible research design
Cleveland & Groenendyk (2010)	No eligible research design
Cleveland & Harris (2010)	No eligible research design
Cleveland et al. (2010a)	No eligible research design
Cleveland (2007)	No eligible research design
Cleveland et al. (2010b)	No eligible research design
Collier (2014)	No eligible intervention
De Miranda (2011)	No eligible research design
Denny (2003)	No eligible intervention
DePue (2015)	No eligible research design
Diehl (2002)	No eligible research design
Doyle (1999)	No eligible intervention
Drake (1989)	No eligible research design
Dusbiber (2006)	No eligible research design
Duszynski (2018)	No eligible research design
Emrich (1981)	No eligible participant population
Eutz (2014)	No eligible research design
Fertman (1989)	No eligible intervention
Finch (2003)	No eligible research design
Finch (2004)	No eligible research design
Finch (2008)	No eligible research design
Finch & Frieden (2014)	No eligible research design
Finch & Karakos (2014)	No eligible research design
Finch et al. (2014)	No eligible research design
Finch (2012)	No eligible research design
Finch (2016)	No eligible research design
Fisher (2015)	Review article
Fleschler (2002)	No eligible intervention
Gibson (1990)	No eligible research design
Gibson (1991)	No eligible research design
Glaude (2016)	No eligible research design
Grana (2010)	No eligible intervention
Grenard (2007)	No eligible intervention
Grunbaum (1993)	No eligible intervention
Grumbaum (2001)	No eligible intervention
Grunbaum (2000)	No eligible intervention
Haardoerfer (2016)	No eligible intervention
Haberle (2014)	No eligible school setting
Harris (2008)	No eligible research design
Harris (2010)	No eligible research design
Harris (2014)	No eligible research design
Hennessy (2015)	No eligible intervention
Higher Education Center (2010)	Review article
Holleran Steiker (2015)	No eligible research design
Horwitz (2000)	No eligible participant population
Hudspeth (2014)	No eligible research design
Hutchison (2017)	No eligible research design
Hymes (2015)	No eligible intervention
Jones (2016)	No eligible research design
Karakos (2014)	No eligible research design
Karakos (2015)	No eligible research design
Karakos et al. (2014)	No eligible research design
Keegan (1996)	No eligible intervention
Kimball (2017)	No eligible research design
Klein (2006)	No eligible research design
Knotts (2018)	No eligible participant population
Knudson (1992)	No eligible intervention
Kochanek (2008)	No eligible research design
Laitman (2014)	No eligible research design
Lanham (2011)	No eligible research design
Lanham & Tirado (2011)	No eligible research design
Laudet (2015)	No eligible research design
Laudet (2013)	No eligible research design
Laudet et al. (2015)	No eligible research design
Laudet (2016)	No eligible research design
Laudet (2014)	No eligible research design
Lin (2017)	No eligible research design
Lincoln (2012)	No eligible research design
Lloyd (2009)	No eligible research design
Lovett (2015)	No eligible research design
Marietti (2015)	No eligible research design
Matto (2014)	No eligible intervention
Misch (2009)	No eligible research design
Moberg (1999)	No eligible research design
Moberg (2008)	No eligible research design
Moberg (1995)	No eligible research design
Moberg (2010)	No eligible research design
Morrison (2006)	No eligible research design
Myrick (2016)	No eligible intervention
Nash (2013)	No eligible research design
Noland (1967)	No eligible research design
Osgood (2012)	No eligible research design
Peters (2003)	No eligible intervention
Preziosi (2017)	No eligible research design
Ramirez (2012)	No eligible research design
Rattermann (2014)	No eligible research design
Riestenberg (2007)	No eligible research design
Russell (2015)	No eligible research design
Russell (2010)	No eligible research design
Russell (2017)	No eligible research design
Scott (2016)	No eligible research design
Shapiro (1981)	No eligible participant population
Shono (2018)	No eligible intervention
Shumway (2013)	No eligible school setting
Shumway (2011)	No eligible research design
Shupp (2015)	No eligible research design
Smagorinsky (1993)	No eligible school setting
Smock (2011)	Review article
Steiker (2014)	No eligible research design
Sussman (2014)	Review article
Taylor (2014)	No eligible research design
Thompson (2014)	No eligible research design
Vogel (2009)	No eligible research design
Vosburg (2016)	No eligible research design
Washburn (2016)	No eligible research design
Watson (2014)	Review article
Webb (2012)	No eligible participant population
Weller (1999)	No eligible intervention
White (2006)	No eligible research design
Wiebe et al. (2010a)	No eligible research design
Wiebe et al. (2010b)	No eligible research design
Wiebe (2018)	No eligible research design
Worfler (2016)	No eligible intervention
Wornson (2013)	No eligible research design
Yule (2018)	No eligible research design
Zheng (2013)	No eligible research design
Zunz (2005)	No eligible research design

### 4.2 RISK OF BIAS IN INCLUDED STUDIES

#### 4.2.1 Recovery high schools

We used the ROBINS‐I tool (Sterne et al., 2016) to assess risk of bias in the one included study (Finch et al., 2018), which used a non‐randomized QED to study the effects of recovery high schools. As specified in the protocol, risk of bias was assessed for four potential confounding factors (prior substance use, prior academic achievement, mental health comorbidities, and readiness to change) and two co‐interventions (self‐help attendance, other substance use counseling services). Risk of bias was assessed separately for the academic outcomes and substance use outcomes; however, given the similarity in measurements and methods across these two outcome domains, the risk of bias ratings were ultimately the same for both of these outcome domains (see Table 5).

**Table 5:** Risk of bias in included studies (ROBINS‐I Tool)

*Finch et al., 2018*			
	**Academic Outcome Domain**	**Substance Use Outcome Domain**	**Comment**
*Bias due to confounding*	Serious risk	Serious risk	There was potential for confounding given students' self‐selection into conditions. The authors used an appropriate analysis method to control for confounders in the domains of prior substance use, prior academic achievement, and psychiatric comorbidities. However, the authors did not control for any confounders in the domain of readiness to change.
*Bias in selection of participants into study*	Low risk	Low risk	There was no indication that any post‐intervention characteristics influenced selection into the study or analysis, and no indication of variability in follow‐up and start of intervention times.
*Bias in classification of interventions*	Moderate risk	Moderate risk	The intervention status of participants was well‐defined, but was determined retrospectively at the end of the study period and not at the start of the study period. However, there is no reason to assume that classification of intervention status could have been affected by knowledge of the outcome (or risk of the outcome).
*Bias due to deviations from intended interventions* *	No information	No information	Not enough information was provided to assess risk of bias in this domain. It is unclear if there were any deviations from the intended intervention.
*Bias due to missing data*	No information	No information	Not enough information was provided to assess risk of bias in this domain. Students lost to follow‐up were excluded from the analysis, and missing data on other variables needed for the analysis were handled using multiple imputation. Attrition rates were not reported separately for the two groups, nor were any reasons for missing data.
*Bias in measurement of outcomes*	Moderate risk	Moderate risk	The methods of outcome assessment were comparable across groups, and any error in outcome measurement is unexpected to be related to intervention status. However, data collectors were not blind to conditions, so it is possible that the outcome measures could have been influenced by knowledge of the intervention condition.
*Bias in selection of reported result*	Low risk	Low risk	There is no registered protocol or analysis plan; however, the outcome measurement and analyses are consistent and clearly defined, and there is no obvious indication of selected analyses or selected subgroup analyses.
*Overall Bias assessment across all domains*	Serious risk	Serious risk	There is serious risk of bias in the confounding domain (due to lack of measurement of the readiness to change confounder), but no critical risks of bias in any other domain.

* Risk of bias assessed based on the effect of assignment to intervention (ITT).

#### 4.2.1.1 Bias due to confounding

The one included study was deemed to have serious risk of bias due to confounding. There was the potential for confounding of the effect of intervention in the study (given that students self‐selected into conditions). Although the authors used an appropriate analysis method to control for confounding factors in the domains of prior substance use, prior academic achievement, and mental health comorbidities, the authors did not control for any measures of students' readiness to change. Per the review protocol, risk of bias due to confounding could occur if the authors did not control for confounders in all four domains.

#### 4.2.1.2 Bias in selection of participants into the study

The one included study was deemed to have low risk of bias in the selection of participants into the study. There was no indication that any post‐intervention characteristics influenced selection into the study or analysis, and no indication that follow‐up or start of intervention times varied for most participants.

#### 4.2.1.3 Bias in classification of interventions

The one included study was deemed to have moderate risk of bias in the classification of interventions. The intervention status of participants was well‐defined, but was determined retrospectively at the end of the study period (i.e., intervention students were those who attended an RHS for at least 28 days). However, there was no reason to assume that classification of intervention status could have been affected by the knowledge of the outcome or the risk of the outcome.

#### 4.2.1.4 Bias due to deviations from intended interventions

The one included study was deemed to have inconclusive risk of bias (no information) due to deviations from intended interventions, assuming the effect of interest is assignment to the intervention. Not enough information was provided to determine whether any deviations from the intended intervention reflected what would be expected in usual educational practice.

#### 4.2.1.5 Bias due to missing data

The one included study was deemed to have inconclusive risk of bias (no information) due to missing data. Participants who were lost to follow‐up during the study period were excluded from the analysis, and missing data on other variables needed for the analysis were handled using multiple imputation. Attrition rates were not reported separately for the two groups, nor were reasons for missing data.

#### 4.2.1.6 Bias in measurement of outcomes

The one included study was deemed to have moderate risk of bias in the measurement of outcomes. The methods of outcome assessment were comparable across groups, and any error in outcome measurement was unexpected to be related to intervention status. However, the outcome assessors were not blinded so it is possible that measures might have been influenced by knowledge of the intervention condition.

#### 4.2.1.7 Bias in selection of the reported result

The one included study was deemed to have low risk of bias in the selection of the reported results. There was no registered protocol or analysis plan; however, the outcome measurement and analyses were consistent and clearly defined, and there was no obvious indication of selected analyses or selected subgroup analyses.

#### 4.2.1.8 Overall risk of bias

Overall, the one included study was deemed to have serious risk of bias. This overall risk of bias disposition resulted from the serious risk of bias assessment in the confounding subdomain (due to a lack of measurement of readiness to change), and no other subdomains with critical risks of bias.

### 4.2.2 Collegiate recovery communities

No eligible studies were identified that examined the effects of CRCs.

## 4.3 SYNTHESIS OF RESULTS

### 4.3.1 Recovery high schools

Only one study met all inclusion criteria for the review (Finch et al., 2018), so we were unable to conduct a meta‐analysis to synthesize findings across multiple studies. Instead we present effect size estimates and corresponding 95% confidence intervals for all eligible outcomes reported in the included study (see Table 6). Future updates to the review will include quantitative syntheses as newly eligible studies become available.

**Table 6:** Effect size estimates and 95% confidence intervals from included studies

*Finch et al., 2018 – Continuous outcomes*				
	**Unadjusted**		**Adjusted**	
**Outcome**	**Hedges' *g***	**95% CI**	**Hedges' *g***	**95% CI**
*Grade point average*	0.65	[0.34, 0.97]	0.26	[‐0.04, 0.56]
*Truancy*	0.16	[‐0.15, 0.46]	0.01	[‐0.29, 0.31]
*Absenteeism*	0.35	[0.05, 0.66]	0.56	[0.25, 0.87]
*Alcohol use*	0.38	[0.08, 0.69]	0.23	[‐0.07, 0.53]
*Marijuana use*	0.62	[0.31, 0.93]	0.51	[0.20, 0.82]
*Other drug use*	0.26	[‐0.05, 0.56]	0.45	[0.14, 0.76]


*Finch et al., 2018 – Binary outcome*				
	**Unadjusted**		**Adjusted**	
**Outcome**	**Odds Ratio**	**95% CI**	**Odds Ratio**	**95% CI**
*Abstinence from alcohol/drugs*	4.17	[0.22, 78.74]	4.36	[1.19 15.98]

*Note*: Adjusted effect size estimates based on intervention effect estimates from regression models that adjusted for pretest scores and propensity scores.

#### 4.3.1.1 Academic performance

The authors reported findings for three outcomes in the academic performance domain: grade point average, truancy, and school absenteeism. All three academic outcomes were collected via self‐report at the study's six‐month follow‐up period. Based on the raw unadjusted outcome data, students in the RHS condition reported higher grade point averages (= 0.65, 95% CI [0.34, 0.97]), lower truancy (= 0.16, 95% CI [‐0.15, 0.46]), and lower absenteeism (= 0.35, 95% CI [0.05, 0.66]) than participants in the comparison condition. However, most of these effect sizes were attenuated when based on the adjusted outcome data from the regression models that controlled for pretest scores and the propensity score: grade point averages (= 0.26, 95% CI [‐0.04, 0.56]), truancy (= 0.01, 95% CI [‐0.29, 0.31]), and absenteeism (= 0.56, 95% CI [0.25, 0.87]). The authors did not report any subgroup findings for participant race/ethnicity, gender, socioeconomic status, mental health comorbidity status, or juvenile justice involvement.

#### 4.3.1.2 Substance use

The authors reported findings for four outcomes in the substance use domain: complete abstinence from alcohol/drugs, frequency of alcohol use, frequency of marijuana use, and frequency of drugs other than alcohol or marijuana. All four substance use outcomes were collected via self‐report at the study's six‐month follow‐up period. Based on the raw unadjusted outcome data, students in the recovery high school condition reported higher rates of abstinence (*OR* = 4.17, 95% CI [0.22, 78.74]), lower alcohol use (= 0.38, 95% CI [0.08, 0.69]), lower marijuana use (= 0.62, 95% CI [0.31, 0.93]), and lower other drug use (= 0.26, 95% CI [‐0.05, 0.56]) than participants in the comparison condition. Again, however, most of these effect sizes were attenuated when based on the adjusted outcome data from the regression models that controlled for pretest scores and the propensity score: abstinence (*OR* = 4.36, 95% CI [1.19, 15.98]), alcohol use (= 0.23, 95% CI [‐0.07, 0.53]), marijuana use (= 0.51, 95% CI [0.20, 0.82]), and other drug use (= 0.45, 95% CI [0.14, 0.76]). The authors did not report any subgroup findings for participant race/ethnicity, gender, socioeconomic status, mental health comorbidity status, or juvenile justice involvement.

#### 4.3.1.2 Substance use related problems

The eligible RHS study did not report any secondary outcomes measuring students' substance use related problems.

### 4.3.2 Collegiate recovery communities

No eligible studies were identified that examined the effects of CRCs on students' academic, substance use, or substance use related problems.

## 5 Discussion

### 5.1 SUMMARY OF MAIN RESULTS

We conducted a systematic literature search to identify all available evidence regarding the effects of recovery high schools (RHSs) and collegiate recovery communities (CRCs) on academic and substance use outcomes among students. Only one identified study met the inclusion criteria for the review (Finch et al., 2018), which examined the effects of RHSs vs. non‐RHSs on the following outcomes: grade point average, truancy, school absenteeism, alcohol use, marijuana use, other drug use, and abstinence from alcohol/drugs. The findings from that study indicated that students in RHSs had significantly lower levels of school absenteeism, marijuana use, and other drug use, and higher rates of abstinence from alcohol/drugs relative to comparison students not enrolled in RHSs. From a practical perspective, for example, these effect sizes translate to 14 fewer days of marijuana use over the past 90 days and five fewer days of school absences for youth in RHSs. When compared to results from a meta‐analysis of outpatient treatment for adolescents (Tanner‐Smith et al., 2016), the effects of recovery schools on substance use outcomes reported in the one study included in this review are stronger than those observed for other types of assertive continuing care programs (0.16, 95% CI [‐0.93, 1.25]). However, there was no evidence that grade point average, truancy, or alcohol use differed for students in RHSs versus the comparison group. These findings must be interpreted with caution, however, given that they are based on only a single study with potentially serious risk of bias due to possible confounding with adolescents' readiness to change.

### 5.2 OVERALL COMPLETENESS AND APPLICABILITY OF EVIDENCE

This review documented the lack of rigorous research evidence regarding the effects of RHS and CRC interventions aimed at promoting academic success and reducing substance use among students in recovery from SUDs. Only one QED study examining RHSs met the inclusion criteria for the review, and no studies were identified that examined the effects of CRCs. Furthermore, no RCTs evaluating the effects of RHSs or CRCs were identified in the review.

#### 5.2.1 Recovery high schools

The one study included in the review examined the effects of RHSs on high school students' outcomes. This study was conducted in three states (Minnesota, Texas, and Wisconsin) in the United States, and thus the findings may not be generalizable to students in other U.S. states or other countries (particularly lower‐ or middle‐income settings). Participants in this study were primarily White high school students, so the findings may not be generalizable to students from different racial/ethnic backgrounds or different ages. The study did not report findings for outcomes related to high school completion, college enrollment, cocaine use, or substance‐related problems; thus there is also no evidence regarding RHS effects for these outcomes. Finally, the study did not report subgroup findings for different types of students, so there is no evidence available regarding variability in effects across types of students.

#### 5.2.2 Collegiate recovery communities

No studies examining the effects of CRCs were identified for inclusion in the review. Thus, there is currently insufficient evidence regarding the effects of CRCs on college students' academic and substance use outcomes.

### 5.3 QUALITY OF THE EVIDENCE

#### 5.3.1 Recovery high schools

The one included study examining RHSs (*n* = 194 students) used a controlled quasi‐experimental design to compare outcomes for students who self‐selected to attend RHSs (or not) after being discharged from substance use treatment. The study was rated as having an overall serious risk of bias due to potential confounding, because participants' readiness to change was not measured or controlled for in the analyses (and readiness to change was identified at the protocol stage as an important confounding factor). Given the lack of evidence from any RCTs, and the serious risk of bias due to potential confounding in the one included QED study, the overall quality of evidence for RHSs is very low.

#### 5.3.2 Collegiate recovery communities

There is insufficient evidence regarding the effects of CRCs. No studies examining the effects of CRCs were identified for inclusion in the review.

### 5.4 LIMITATIONS AND POTENTIAL BIASES IN THE REVIEW PROCESS

The main limitation of this review is the insufficient evidence base regarding the effects of recovery schools: only one study examining RHSs and no studies examining CRCs met the inclusion criteria. Given the limited evidence available, it is premature to draw any firm conclusions regarding the effects of recovery schools on the academic and substance use outcomes for students in recovery from substance use.

This review followed current methodological guidance from the Campbell Collaboration for the conduct and reporting of intervention effectiveness reviews (2014a, 2014b). We conducted a comprehensive and systematic literature search aimed at identifying all relevant literature, used two independent reviewers for screening and data extraction, and followed all procedures outlined in our review protocol—thereby minimizing any potential biases during the systematic review process.

Nonetheless, it is possible that some relevant studies may not have been identified in the literature search. The one included study was an unpublished manuscript obtained from our email contact with experts (although several of the supplementary reports from this study were identified in the electronic literature search). Given the paucity of literature on RHSs and CRCs, and the fact that the one included study was unpublished, it is possible that we failed to identify other unpublished studies that met our inclusion criteria. This potential limitation highlights the importance of conducting extensive supplementary (grey) literature searches when seeking evidence on the effects of recovery schools.

### 5.5 AGREEMENTS AND DISAGREEMENTS WITH OTHER REVIEWS

The findings from the current review, based on results from one primary study, suggest that RHSs may have beneficial effects on U.S. high school students' academic and substance use outcomes. The review yielded insufficient evidence regarding the effects of CRCs on students' outcomes. To date, we are aware of no other systematic reviews or meta‐analyses that have synthesized the empirical evidence on the effects of recovery schools. Several narrative reviews have highlighted the potential promise of RHSs or CRCs (Ashford, Brown, Eisenhart, Thompson‐Heller, & Curtis, 2018; Fisher, 2014; Smock et al., 2011; Watson, 2014), but none of these reviews reported effect sizes from the included studies.

## 6 Authors' conclusions

There is insufficient evidence regarding the effectiveness of RHSs and CRCs for improving academic and substance use outcomes among students in recovery from SUDs. Only one identified study examined the effectiveness of RHSs. Although the study reported some beneficial effects, the results must be interpreted with caution given the study's potential risk of bias due to confounding and limited external validity. No identified studies examined the effectiveness of CRCs across the outcomes of interest in this review, so it is unclear what effects these programs may have on students.

### 6.1 IMPLICATIONS FOR PRACTICE AND POLICY

As evidenced by recent enthusiasm for education‐based supports for youth in recovery (NIDA, 2014; ONDCP, 2014; White, 2009), RHSs and CRCs may offer intuitive and theoretical appeal to many educational practitioners and policymakers. However, the paucity of rigorous evidence on the effectiveness of recovery schools, as documented in this review, suggests the need for caution in the widespread adoption of recovery schools for students in recovery from SUDs. One U.S. study (Finch et al., 2018) reported potential beneficial effects of RHSs on students' outcomes, and reported no evidence of any harmful or adverse effects. School administrators and educational policymakers may thus want to consider whether RHSs may be feasible and acceptable to implement in their communities. It is premature, however, to suggest any widespread implementation of CRCs, given the lack of rigorous evidence regarding their effectiveness.

### 6.2 IMPLICATIONS FOR RESEARCH

Given the lack of empirical support regarding the effectiveness of recovery schools, additional rigorous evaluation studies are needed. This includes evaluations of CRCs and evaluations of RHSs that aim to replicate and expand upon the findings from the one study included in the review. It is critical for future research studies to use experimental and strong quasi‐experimental research designs that will permit causal inferences regarding the effectiveness of recovery schools for promoting the well‐being of students in recovery from SUDs. Although studies using pre‐experimental, single‐case, or qualitative designs can provide valuable information on recovery schools (e.g., for a review of the qualitative literature on CRCs see Ashford et al., 2018), there is a clear need for controlled evaluation studies that will permit causal inferences regarding program effects. Future controlled trials examining the effectiveness of recovery schools should also report effects across key subgroups of students (i.e., based on age, race/ethnicity, baseline substance use, baseline academic performance, psychiatric comorbidities, and juvenile justice involvement), to help identify the types of youth who benefit most (or least) from participating in recovery schools. Finally, additional economic research is needed to examine the financial costs and cost savings associated with recovery schools, which will help school administrators by providing evidence regarding the potential cost‐benefits associated with recovery schools.

## Information about this review

### Review authors

**Lead review author**

**Table:** 

**Name:**	**Emily A. Hennessy**
Title:	MPhil, PhD
Affiliation:	*Current:* Institute for Collaboration on Health, Intervention and Policy University of Connecticut *Former:* Peabody Research Institute, Human and Organizational Development Vanderbilt University, Peabody College
Address:	2006 Hillside Road
City, State, Province or County:	Storrs, CT
Postal Code:	06269‐1248
Country:	USA
Phone:	978‐810‐0398
Email:	Emily.Hennessy@uconn.edu
City, State, Province or County:	Storrs, CT
**Co‐authors**	
**Name:**	**Emily E. Tanner‐Smith**
Title:	Associate Professor
Affiliation:	*Current:* University of Oregon Counseling Psychology and Human Services Educational Methodology, Policy, and Leadership *Former:* Peabody Research Institute, Human and Organizational Development Vanderbilt University
Address:	5251 University of Oregon
City, State, Province or County:	Eugene, OR
Postal Code:	97403‐5251
Country:	USA
Phone:	541‐346‐2365
Email:	etanners@uoregon.edu
**Name:**	**Andrew J. Finch**
Title:	Associate Professor of the Practice
Affiliation:	Vanderbilt University, Human and Organizational Development
Address:	Box 90 GPC, 230 Appleton Place
City, State, Province or County:	Nashville, TN
Postal Code:	37203
Country:	USA
Phone:	615‐322‐8684
Email:	Andrew.j.finch@vanderbilt.edu
**Name:**	**Nila A. Sathe**
Title:	Director, Medical Evidence
Affiliation:	Premier Inc.
Address:	13034 Ballantyne Corporate Place
City, State, Province or County:	Charlotte, NC
Post code:	28277
Country:	USA
Phone:	615‐525‐6873
Email:	Nila_sathe@premierinc.com
**Name:**	**Shannon A. Kugley**
Title:	Researcher
Affiliation:	Chapin Hall at the University of Chicago
Address:	1313 East 60th Street,
City, State, Province or County:	Chicago, Illinois
Post code:	60637
Country:	United States
Phone:	615‐351‐4461
Email:	skugley@chapinhall.org

### Roles and responsibilities

- Content: Finch, Hennessy, Tanner‐Smith
- Systematic review methods: Hennessy, Kugley, Sathe, Tanner‐Smith
- Statistical analysis: Hennessy, Tanner‐Smith
- Information retrieval: Hennessy, Kugley, Sathe

### Sources of support

This review was supported with grant CSR1.07 from the Campbell Collaboration. The opinions expressed are those of the authors and do not necessarily represent the views of the Campbell Collaboration.

### Declarations of interest

One of the authors (AJF) is a non‐voting unpaid board member for the Association of Recovery Schools, but he will receive no financial benefit from the findings published in this review. Three of the authors of this review (AJF, EAH, ETS) are co‐authors on the one primary study included in the review. Therefore, these three authors were not involved in the data extraction for that study; external and independent data collectors were used to extract all data from that study.

The authors of this review have no other conflicts of interest to declare.

### Plans for updating the review

The lead reviewer anticipates updating the review on a five‐year cycle, pending continued research on the topic.
